# The Effect of Iodine-Containing Nano-Micelles, FS-1, on Antibiotic Resistance, Gene Expression and Epigenetic Modifications in the Genome of Multidrug Resistant MRSA Strain *Staphylococcus aureus* ATCC BAA-39

**DOI:** 10.3389/fmicb.2020.581660

**Published:** 2020-10-22

**Authors:** Oleg N. Reva, Ilya S. Korotetskiy, Monique Joubert, Sergey V. Shilov, Ardak B. Jumagaziyeva, Natalya A. Suldina, Alexandr I. Ilin

**Affiliations:** ^1^Centre for Bioinformatics and Computational Biology (CBCB), Department of Biochemistry, Genetics and Microbiology, University of Pretoria, Pretoria, South Africa; ^2^Scientific Center for Anti-Infectious Drugs (SCAID), Almaty, Kazakhstan

**Keywords:** *Staphylococcus aureus*, MRSA, transcriptomics, genomics, epigenetics, antibiotic resistance reversion, iodine, FS-1

## Abstract

Application of supplementary drugs which increase susceptibility of pathogenic bacteria to antibiotics is a promising yet unexplored approach to overcome the global problem of multidrug-resistant infections. The discovery of a new drug, an iodine-containing nano-molecular complex FS-1, which has proven to improve susceptibility to antibiotics in various pathogens, including MRSA strain *Staphylococcus aureus* ATCC BAA-39^TM^, allowed studying this phenomenon. Chromosomal DNA and total RNA samples extracted from the FS-1 treated strain (FS) and from the negative control (NC) cultures were sequenced by PacBio SMRT and Ion Torrent technologies, respectively. PacBio DNA reads were used to assemble chromosomal DNA of the NC and FS variants of *S. aureus* BAA-39 and to perform profiling of epigenetically modified nucleotides. Results of transcriptional profiling, variant calling and detection of epigenetic modifications in the FS variant were compared to the NC variant. Additionally, the genetic alterations caused by the treatment of *S. aureus* BAA-39 with FS-1 were compared to the results of a similar experiment conducted with another model organism, *E. coli* ATCC BAA-196. Several commonalities in responses of these phylogenetically distant microorganisms to the treatment with FS-1 were discovered, which included metabolic transition toward anaerobiosis and oxidative/osmotic stress response. *S. aureus* culture appeared to be more sensitive to FS-1 due to a higher penetrability of cells by iodine bound compounds, which caused carbonyl stress associated with nucleotide damaging by FS-1, abnormal epigenetic modifications and an increased rate of mutations. It was hypothesized that the disrupted pattern of adenine methylated loci within methicillin-resistance chromosome cassettes (SCC*mec*) may promote excision of this antibiotic resistance determinant from chromosomes while the altered pattern of cytosine methylation was behind the adaptive gene regulation in the culture FS. The selection against the antibiotic resistance in bacterial populations caused by abnormal epigenetic modifications exemplifies possible mechanisms of antibiotic resistance reversion induced by iodine-containing compounds. These finding will facilitate development of therapeutic agents against multidrug-resistant infections.

## Introduction

The misuse of antibiotics, as well as inappropriate prescription and overuse, has led to strong selective pressure, resulting in the survival and wide distribution of drug-resistant pathogens that threaten the public health system globally (Barbosa and Levy, 2000; Ventola, 2015). Acquired drug resistance to antimicrobial agents has been a widely recognized problem (Tenover, 2001; Spellberg et al., 2004; Wall, 2019; Rocha-Granados et al., 2020). Bacteria can respond rapidly to environmental changes owing to their short growth cycles, which enable them to evolve and adapt rapidly and survive under unfavorable conditions (Clatworthy et al., 2007). Currently, the rate of resistant bacteria is increasing, whereas the development of new antibiotics has dramatically declined over the past two decades (Spellberg et al., 2004).

Various factors are involved in the emergence and spread of drug resistant bacteria. These factors include mutations, which can modify target proteins; the transfer of genetic material, known as horizontal gene transfer; selective pressure in healthcare and community settings, which facilitates the development and distribution of multiple resistant bacteria; and in some cases, inability to detect emerging resistance phenotypes (Wang et al., 2017; Kumar et al., 2019). Resistance is generally associated with a decrease in bacterial fitness. It was expected that the physiological cost for bacteria to maintain resistance genes in the absence of antibiotics would be large enough to select substantially against the drug resistance. However, this has not been generally observed for various reasons. Firstly, the fitness cost for bacteria to maintain resistance is not always large enough to select for loss of the resistance alleles; therefore, even after removal of the drug, resistance may remain in the population for an extended period (Sjolund et al., 2003; Andersson and Hughes, 2010). Compensatory mutations and/or genetic regulatory mechanisms can also compensate for the large fitness cost of resistance by only activating resistance in the presence of the drug (Durão et al., 2018; Lin et al., 2018). Lastly, resistance mutations may provide the resistant strains with a fitness advantage by conferring increased virulence (Roux et al., 2015).

With the increasing occurrence of multidrug-resistant bacteria, monotherapy treatment is gradually becoming less adequate, necessitating the use of drug combination therapies (Nigam et al., 2014; Zhu et al., 2014). Therefore, it is of great importance to devise strategies that focus on the application of supplementary drugs to increase susceptibility to regular antibiotics by inhibiting bacterial growth while reversing the selection for resistance. Strategies to combat antibiotic resistance with combination drug therapy have been proven promising since the late 1940s. Co-administration of streptomycin and para-aminosalicylic acid showed reduced evolution of resistant *Mycobacterium tuberculosis* strains (Dunner et al., 1949). Drug combinations are also currently being used in most treatments of infectious diseases (Poulikakos et al., 2014). Drug-induced reversion of antibiotic-resistant pathogens into sensitive phenotypes is a prospective approach to target the mechanisms and evolution of bacterial resistance (Baym et al., 2016; Ilin et al., 2017; van Niekerk et al., 2018).

Various mechanisms that reduce or invert the selective advantage of antibiotic resistance have previously been studied. Administering antibiotics with chemicals that inhibit specific resistance mechanisms is generally accepted as the best-established approach to neutralize the evolutionary advantages of resistant strains. However, this does not necessarily create a competitive disadvantage and the relative prevalence of resistance within a bacterial population is not reduced. Negative selective pressure is required to reduce resistant strains in the population, even when the antibiotic is present (Sjolund et al., 2003, 2005). More recent studies focused on using the evolutionary and physiological interactions between drugs not only to neutralize the selective advantage of resistant strains, but also to select against them actively by imposing a direct cost on resistance. According to Baym et al. (2016), combinations of drugs can interact to impose a direct cost on resistance in three ways. Firstly, co-administrated drugs can cause suppressive interactions, where when drug A suppresses drug B, bacteria that became resistant to drug A can be inhibited more strongly by drug B owing to loss of their protective effect. This causes the concentration of bacterial growth to be non-monotonic, and sensitive bacteria grow better than resistant bacteria at high concentrations of drug B when drug A is present. Secondly, co-administered drugs can interact synergistically, creating a concentration regime where sensitive bacteria can continue to grow, while the growth of resistant strains is inhibited. Thirdly, resistance to drug A can increase the efficacy of drug B owing to evolutionary trade-offs, allowing selection against resistant strains even when drug A is present (Baym et al., 2016).

Key drivers of evolution are mutations of an organism’s DNA, which generate genetic variation (Hershberg, 2015). Bacteria are constantly faced with the challenge to maintain fitness in changing environments, subjecting them to various stresses, which include oxidative stress and DNA damage. In response to these stresses, bacteria alter their phenotypes by modulating gene expression levels. For a long time, it was generally believed that adaptable variations in bacterial populations are possible only through mutational processes. The role of mutations in heritable phase variations of bacterial populations is a certainty. Bacteria have phase-variable genes, referred to as contingency genes, that are highly mutable compared to housekeeping genes (Phillips et al., 2019a,b). Slipped-strand mispairing (SSM) is described as a mechanism that promotes phase variation by reversible mispairing of short repeat sequences between mother and daughter strands during DNA replication (Henderson et al., 1999; Low and Casadesús, 2008; Harhay et al., 2019). SSM is not the only mechanism of phase variation. There are other mechanisms involving homologous recombination, insertion/excision of transposons and site-specific recombination. Recently, the role of differential DNA methylation and other epigenetic mechanisms of phase variations enabling the adaptation of bacterial populations to harsh or changing environments including antibiotic treatment regiments were pointed out (van der Woude and Bäumler, 2004; Sánchez-Romero and Casadesús, 2020). DNA methylation enables bacteria to control the reversible ON/OFF switching of important genes epigenetically. An example of this includes the pyelonephritis-associated pili (*pap*) operon in uropathogenic *E. coli* controlled by DNA adenine methylase (Dam). Switching between the ON/OFF states of the *papBA* genes determines the binding of two proteins at two GATC sites, before and after the promoter. The operon is turned to the ON state when methylation occurs proximal to the promoter, and vice versa (Hernday et al., 2002; van der Woude and Bäumler, 2004). Another example includes the gene *flu*, encoding for the outer membrane protein Ag43 in *E. coli*. This gene is regulated at three GATC sites and its expression is repressed by the oxidative stress response protein, OxyR. Binding of OxyR to a GATC site masks this site, therefore blocking methylation by Dam, and turning expression to the OFF state (Haagmans and van der Woude, 2000). Other study showed that Dam methylases can modulate pathogenicity of *Klebsiella pneumoniae* (Fang et al., 2017) and regulate the expression of drug resistance related genes including multidrug efflux pumps that leads to a fast acquisition by *E. coli* of a resistance to various antibiotics through epigenetic DNA modifications (Adam et al., 2008). Application of PacBio SMRT sequencing demonstrated a crucial role of directed DNA methylation in micro-evolution of the outbreak clones of methicillin-resistant *Staphylococcus aureus* (MRSA) leading to attenuation of *agr* transcriptional regulator expression and upregulation of genes involved in stress response and biofilm formation (Sullivan et al., 2019).

As epigenetic modifications are so important for the virulence and antibiotic resistance of pathogenic bacteria, the possibility of using the phase variation processes and epigenetic modifications to induce and direct antibiotic resistance reversion in populations of multidrug resistant pathogens may be assumed; however, it has never been explored. Recently, a new medicine, FS-1, was introduced into clinical practice as a supplement to antibiotic therapy for drug-resistant tuberculosis (Ilin and Kulmanov, 2014; Ilin et al., 2017; Korotetskiy et al., 2017; Islamov et al., 2018; van Niekerk et al., 2018). FS-1 is an iodine-containing nano-micelle, which reverses the susceptibility of resistant pathogens to conventional antibiotics. Sequencing of extensively drug-resistant strains of *M. tuberculosis* (XDR TB), which were reverted to the sensitive phenotype by treatment with FS-1, showed no mutations to explain this phenomenon (Ilin et al., 2015, 2017). Epigenetic mechanisms of antibiotic resistance reversion were hypothesized. Later, the antibiotic resistance reversion was reproduced on beta-lactam antibiotic resistant strain *Escherichia coli* ATCC BAA-196^TM^ (Korotetskiy et al., 2019a) and MRSA *Staphylococcus aureus* ATCC BAA-39^TM^ (Joubert et al., 2019), which are more suitable for laboratory experiments than XDR TB.

*Staphylococcus aureus* is a representative of Gram-positive bacterial human pathogens, responsible for a wide variety of clinical manifestations. The occurrence of antibiotic resistance in *S. aureus* has been studied widely in view of their ability to develop resistance to nearly any antibiotic (Chambers and Deleo, 2009), exemplifying the adaptive evolution of bacteria during the antibiotic era. *S. aureus* is a fast-growing bacterium and proper growth can easily be maintained in a laboratory, making it simple and inexpensive to work with. This makes *S. aureus* a desirable model for studying the evolution of drug resistance and establishing new approaches to combat the resistance by induced antibiotic resistance reversion. The aim of this research was to study antibiotic resistance phenotype, transcriptome and epigenetic modifications in the genome of multidrug-resistant *Staphylococcus aureus* ATCC BAA-39 cultivated with a sublethal concentration of the drug FS-1. Additionally, the differential gene expression and epigenetic modifications in FS-1 treated *S. aureus* ATCC BAA-39 were compared to the results obtained on the phylogenetically distant model organism, *E. coli* ATCC BAA-196 (Korotetskiy et al., 2020b).

## Materials and Methods

### Bacterial Cultures

The model multidrug-resistant microorganism *Staphylococcus aureus* ATCC BAA-39^TM^ was obtained from the ATCC collection and kept in a freezer at −80°C. Bacteria were cultivated on Mueller-Hinton (MH) liquid or solid media (Himedia, India) (Vitko and Richardson, 2013).

### Antibiotic Resistance Reversion

**FIGURE 1:**
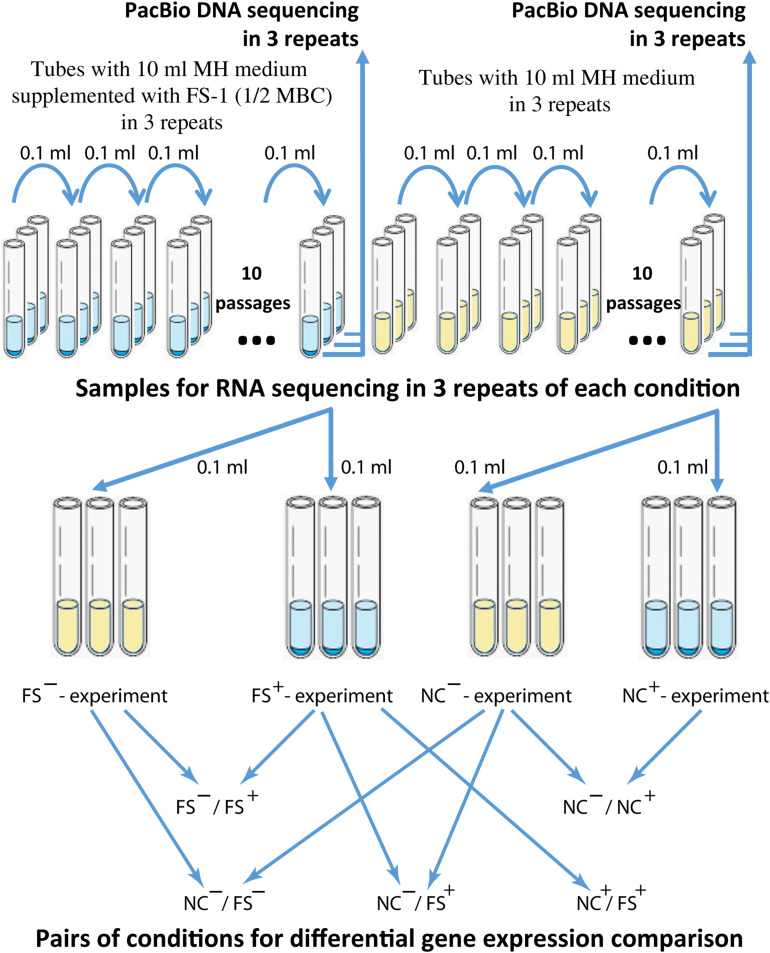
The scheme of the antibiotic resistance reversion experiment. Different experiments were denoted as (FS^–^) – FS-1 treated culture grown for RNA extraction on MH medium without the drug FS-1; (FS^+^) – FS-1 treated culture grown on MH supplemented with 450 μg/ml; (NC^+^) – negative control culture grown on MH supplemented with 450 μg/ml; (NC^–^) – negative control culture grown on MH without the drug FS-1.

Bacteria were inoculated into test-tubes with 10 ml of liquid MH medium supplemented with FS-1 (450 μg/ml) that corresponds to 1/2 minimal bactericidal concentration (MBC) of the drug estimated for the strain *S. aureus* BAA-39. As the negative control, the culture was cultivated in the same medium without the drug. Test-tubes were incubated at 37°C for 24 h and then 0.1 ml aliquots of the cultures (in average 1.2 × 10^9^ CFU/ml, no significant difference in the optical density of the overnight NC and FS cultures was observed) were transferred to fresh tubes with the corresponding media. After 10 passages, the experimental and control bacteria were cross-inoculated in three repetitions into tubes with drug-containing and drug-free media for further overnight incubation (24 h), followed by DNA and RNA extraction. The scheme of the experiment is shown in Figure 1. The susceptibility of bacterial cultures was evaluated by measuring the growth inhibition zones around disks (Himedia, India) with the following antibiotics: amoxicillin, clindamycin, gentamicin, methicillin, oxacillin, imipenem, tobramycin, cefamandole and erythromycin. The bacterial cultures were denoted as susceptible (S), intermediate (I) or resistant (R) by comparing the experimental results with the threshold inhibition zones recommended for the given antibiotics in the CLSI (2010) and by Sarker et al. (2014).

### Radiological Study of FS-1 Complexing With Chromosomal DNA

To carry out the radiological study, isotope ^131^I was used for the synthesis of FS-1. The documented radioactivity of the isotope 20 MBq/ml was controlled at the beginning of the synthesis. Radioactivity was measured by β-spectrometer Hidex 300 SL (Finland).

Bacteria were incubated in a thermo-shaker overnight (24 h) on MH medium at 37°C. The culture growth was centrifuged at 5,000 *g* and the cells were resuspended in saline to achieve 1 × 10^9^ CFU/ml. Aliquots of 0.4 ml of the marked FS-1 solution (total radioactivity of an aliquot was 270 Kbq) were added to 7.6 ml of the bacterial suspension and rigorously shaken. Samples were incubated for 1 h at 37°C in a thermo-shaker, Thermomixer comfort (Germany). After incubation, bacterial cells were washed from FS-1 by three rounds of centrifugation at 5,000 *g* and re-suspended in sterile saline. DNA samples were extracted from bacterial cells using PureLink Genomic DNA Kits (Publication Number: MAN0000601, Revision 2.0), following the manufacturer’s recommendations. DNA purity and concentration were controlled by NanoDrop 2000C (United States) at wavelengths 260 and 280 nm. Then the DNA samples were resolved in scintillation solute (ULTIMA GOLD LLT, PerkinElmer) to a final volume of 5 ml. The residual radiation was measured by β-spectrometer Hidex 300 SL (Finland).

### DNA Extraction and PacBio Sequencing

DNA samples were extracted from bacterial cells using PureLink Genomic DNA Kits (Thermo Fisher, MAN0000601, Revision 2.0) following the manufacturer’s recommendations.

Samples were prepared in three repetitions according to a guide of preparation of SMRT bell templates for sequencing on the PacBio RS II System. The samples were sequenced in Macrogene (South Korea) on SMRT Cell 8Pac V3 cells using the DNA Polymerase Binding Kit P6 and DNA Sequencing Reagent 4.0 v2 following the SMR Tbell 20-kb library preparation protocol. The raw PacBio reads are available at NCBI SRA database under accession numbers SRX5852332 (run SRR9077059) and SRX5843551 (run SRR9067444) for NC and FS genomes, respectively.

### Total RNA Extraction and Ion Torrent Sequencing

Isolation of total RNA was performed with the RiboPure Bacteria Kit (Ambion, Lithuania) according to the developer’s guidelines. The quality and quantity of the resulting RNA were determined using the Qubit 2.0 Fluorometer (Thermo Scientific, United States) and Qubit RNA HS Assay Kit (Invitrogen, United States). Purification of total RNA from ribosomal RNA was carried out using the MICROBExpress Bacterial mRNA Purification Kit (Ambion, Lithuania), as recommended by the developer. The efficiency of the template-RNA purification was determined on the Bioanalyzer 2100 (Agilent, Germany) with the RNA 6000 Nano LabChip Kit (Agilent Technologies, Lithuania).

The RNA fragment library was prepared by enzymatic fragmentation with the Ion Total RNA Seq Kit V2 (Life Technologies, United States). Barcoding of the formed library was carried out with the Ion Xpress RNA-Seq Barcode 01-16 Kit (Life Technologies, United States), according to the manufacturer’s instructions. RNA sequencing was performed in three repetitions for each condition using the Ion Torrent PGM sequencer (Life Technologies, United States) with the Ion 318 Chip Kit V2. Resulted 12 sets of the raw RNA reads (NC and FS variants cultivated on MH and MH with 450 μg/ml FS-1 in three repetitions, see Figure 1) were deposited at NCBI SRA database under accession numbers SRR10728551-SRR10728554.

Reads obtained were demultiplexed with FASTQCMCF prior to being trimmed on quality (Phred score = 21) using the Raw RNA-Seq Data Processing pipeline implemented in Unipro UGENE v35.1 (Golosova et al., 2014). Reads shorter than 30 bp were filtered out.

### Genome Assembly and Annotation

The raw PacBio reads were converted into XML subreads by the function HdfSubreadSets of the SMRT Link v5.0.1 software package using the parameters set by default. The complete genome assembly was performed using *polished_falcon_fat* pipeline, available from the SMRT Link v5.0.1 software (Chin et al., 2013; Roberts et al., 2013). Two genome variants, NC and FS, were obtained as single contig sequences without gaps and ambiguities with the respective coverage of 966 and 906 overlapping reads per nucleotide. The completeness of the final assemblies was evaluated using the benchmarking universal single-copy orthologous (BUSCO) software (Simão et al., 2015). Genome annotation was performed using the RAST Server^1^ (Aziz et al., 2008) and then manually corrected. The locations of horizontally transferred genomic islands were identified by the program SeqWord Genome Island Sniffer (Bezuidt et al., 2009). The same program was used to identify the replication origin and terminus on bacterial chromosomes by GC-skew between the leading and lagging strands (Freeman et al., 1998). Antibiotic resistance genes were predicted using CARD-RGI Web-based tool^2^ (Alcock et al., 2020). This research project was registered in the BioProject database at NCBI under the accession number PRJNA480363. PacBio reads generated for this study are available from the BioProject Web-site. The assembled and annotated genomes of the variants NC and FS of *Staphylococcus aureus* ATCC BAA-39^TM^ were published in NCBI GenBank under the accession numbers CP033505 and CP033506, respectively.

### Gene Expression Profiling

The differential expression was done using the R-3.4.4 software. Firstly, a reference index was built for each reference genome using the *buildindex* function available in the *Rsubreads* package (Bioconducter). The obtained RNA fragments were aligned to the relevant reference genome with the use of the function *align*. The aligned BAM files and relevant GFF annotation files were then used as input for the *featureCounts* function to obtain gene counts. The R packages DESeq2 (Bioconducter) and *GenomicFeatures* was then used for the differential expression analyses (Love et al., 2014). The DESeq2 algorithm normalizes read counts by gene lengths and by total number of reads in samples. Grouping of co-expressed genes was performed by using Principle Coordinates Analysis (PCoA) algorithm implemented in the program PAleontological STatistics (PAST) 4.02^3^ (Hammer et al., 2001). Downstream networks of regulated genes were constructed using the Web based tool PheNetic (De Maeyer et al., 2013) based on the regulation network available from the PheNetic Web site, which was designed for the strain *S. aureus* XN108 (CP007447.1) (Zhang et al., 2014). Pairs of homologous genes in the genomes *S. aureus* BAA-39 and *E. coli* BAA196 were identified using the program GET_HOMOLOGUES (Contreras-Moreira and Vinuesa, 2013).

### Profiling of Epigenetic Modifications

PacBio sequencing allows the prediction of epigenetically modified bases by using the base call kinetics analysis, a useful technique for base modification profiling of bacterial genomes (Liu et al., 2019). Processing of modified bases by SMRT sequencing technologies requires a longer time than the average. The time of base calling may vary sporadically in a rather wide range. The program accounts for repeated base call delays termed interpulse duration (IPD) in overlapping reads to score the likelihood of the epigenetic modification at a given site that is estimated as base modification (BM) score. Tools available in the SMRT Link v5.0.1 software were used with an in-house Python script to generate a pipeline to perform base call kinetic analysis on the PacBio reads generated from chromosomal DNA (Joubert et al., 2019). The pipeline consists of the following steps: (i) the complete genome consensus sequences in FASTA format, obtained by assembling PacBio reads, were indexed by the program *samtools* to be used as the reference sequence for PacBio read alignment; (ii) PacBio reads were converted from the original BAX.H5 format to BAM format by the tool *bax2bam*; (iii) reads stored in BAM files were aligned against the indexed reference sequence by the tool *blasr* (Chaisson and Tesler, 2012); (iv) aligned reads in BAM format were sorted by locations and indexed by *samtools sort* and *index* functions; and (v) sorted and indexed BAM files were analyzed by the tool *ipdSummary* to evaluate the base call kinetics for every nucleotide in the reference genome (the output file *^∗^_kinetics.csv*). The program stores all the estimated parameters together with context sequences into an output file *^∗^_basemods.gff*. In step (vi), contextual motifs of base modifications were searched by the tool *motifMaker*. Thereafter, the epigenetic profiles of the studied genomes were visualized using an in-house Python script, which uses the *^∗^_kinetics.csv* and *^∗^_basemods.gff* output files. This in-house pipeline was created to allow a detailed analysis of intermediate files. To check the correctness of predictions by the in-house pipeline, the standard SMRT Link DNA modification prediction protocol, *ds_modification_motif_analysis* described in the paper by O’Dell et al. (2018), was used also.

In addition to scoring each nucleotide, the program analyses the context information around the modified bases and identifies contextual motifs. The most common type of epigenetic modification is the methylation of adenine (m6A) and cytosine (m4C) residues. However, this analysis identifies many other nucleotides delaying SMRT sequencing owing to modifications of unknown nature. Thereafter, in the text, these unknown modifications are denoted modA, modG, modC and modT.

### Statistical Analysis

All measurements were performed at least 3 times. Average values and standard deviations were used for comparison. To evaluate patterns of gene expression at an experimental condition compared to the control condition, RNA samples were isolated in three repetitions. DESeq2 algorithm estimates fold change and *p*-values for every gene. Genes with two-fold or above differential expression and *p*-value smaller than 0.05 were considered as regulated.

SMRT Link v5.0.1 DNA modification pipeline identifies nucleotide positions with a significant base call delay repeated in multiple overlapped reads. The program calculates several statistical parameters such as the interpulse duration (IPD) ratio of the average base call time to the expectation; and the base modification (BM) scores. BM scores are phred-transformed *p*-values to validate that a kinetic deviation existing at this base position is statistically reliable. BM scores 14 and 21 correspond respectively to *p*-values 0.05 and 0.01. Only those bases that gained BM scores above 20 in all three repetitions were taken into consideration. As in this study, NC and FS variants were sequenced in three repetitions, an additional level of validation of epigenetic modifications was implemented using the in-house Python 2.7 script, which selected only those modifications predicted in all three sets of PacBio reads. Average BM scores were calculated for the selected sites.

Pearson correlation coefficients (*C*_*p*_) of gene co-expression were calculated by Eq. 1 implemented in an in-house Python 2.7 script.

(1) $$\begin{array}{c} C_{P}=\frac{N\,\sum x_{i}\,y_{i}-\sum x_{i}\,\sum y_{i}}{\sqrt{\left(N\,\sum x_{i}^{2}-{\left(\sum x_{i}\right)}^{2}\right)\,\left(N\,\sum y_{i}^{2}-{\left(\sum y_{i}\right)}^{2}\right)}} \end{array}$$

where *x*_*i*_ and *y*_*i*_ are fold-change values estimated for every *i*’s homologous gene, *N* – total number of compared homologous genes shared by two genomes.

## Results

### Antibiotic Resistance Reversion

Antibiotic resistance reversion induced by FS-1 was evaluated after cultivation of the multidrug-resistant bacterium for 10 days on the medium containing a sub-lethal 1/2 MBC of FS-1 with daily re-inoculation into fresh medium. The negative control cultures were cultivated in the same medium without FS-1. The scheme of the experiment is shown in Figure 1. Susceptibility of *S. aureus* to antibiotics before the experiment and after cultivation at experimental (FS) and negative control (NC) conditions was detected by measuring the growth inhibition zones around antibiotic-containing disks in three repetitions (Table 1).

**TABLE 1:** Antimicrobial susceptibility characteristics of *Staphylococcus aureus* BAA-39 by disk-diffusion assay.

Antibiotic	Initial culture		NC after 10 passages		FS after 10 passages	
	Inhibition zone (mm)*	S/I/R^†^	Inhibition zone (mm)	S/I/R	Inhibition zone (mm)	S/I/R
Amoxicillin (30 μg)	10 ± 0	R	12 ± 2.8	R	40 ± 0	S
Clindamycin (10 μg)	0	R	0	R	24 ± 9.5	S
Gentamicin (10 μg)	6.66 ± 0.94	R	8 ± 2.9	R	25 ± 0	S
Methicillin (30 μg)	9.7 ± 0.58	R	23 ± 2.9	S	34 ± 1.2	S
Oxacillin (1 μg)	0	R	12 ± 0	I	18 ± 5,19	S
Imipenem (10 μg)	37 ± 1.7	S	43 ± 2.9	S	50 ± 0	S
Tobramycin (10 μg)	11 ± 0.6	R	11 ± 1.2	R	27 ± 2.5	S
Cefamandole (30 μg)	17 ± 0.6	I	20 ± 0	S	17 ± 0.6	I
Erythromycin (15 μg)	0	R	0	R	24 ± 1.2	S

*Inhibition zones were measured in 3 repetitions and the standard deviation was calculated; ^†^S – susceptible; I – intermediate; R – resistance response as defined in the Performance Standards for Antimicrobial Disk Susceptibility Test (2010) and by Sarker et al. (2014).

The initial *S. aureus* BAA-39 strain was resistant to many tested antibiotics, except for imipenem and cefamandole (Table 1). The cultivation of multidrug-resistant bacteria on the medium without antibiotics was favorable for selecting more sensitive variants. As a result, after 10 passages on the regular medium, the culture gained susceptibility to methicillin and oxacillin. Rapid decline of bacterial drug resistance on the antibiotic-free medium due to the fitness cost associated with drug resistance mechanisms was described previously. Mechanisms of drug resistance decline involves accumulation of mutations reducing the fitness cost, which, due to absence of antibiotics in the medium, reduced drug resistance (Dunai et al., 2019). Other authors suggested epistatic mechanisms of regulation of the level of antibiotic resistance and the associated fitness cost (Durão et al., 2018). The significant methicillin resistance reduction observed in the culture NC may be explained also by excision of staphylococcal methicillin-resistance chromosome cassettes (SCC*mec*) from chromosomes that occurs naturally on the methicillin-free media with a rate from 10^–8^ to 10^–5^ depending on the tested strain and the presence of other genomic islands (Stojanov et al., 2015; Almebairik et al., 2020). Cultivation with 1/2 MBC FS-1 induced antibiotic resistance reversion in the treated bacterium compared to the initial strain and the variant NC. After 10 passages on the medium with FS-1, the initially resistant *S. aureus* BAA-39 strain became susceptible to all the tested antibiotics except for cefamandole (Table 1).

### Complete Genome Assembly

DNA reads, generated from genomes of the NC and FS variants of *S. aureus* ATCC BAA-39, were assembled into single contig sequences of bacterial chromosomes with no gaps or ambiguities. No plasmids were identified in these genomes. The lengths of the NC and FS genomes were 2,791,218 bp and 2,792,888 bp, respectively. The completeness of the final assemblies was considered satisfactory, as revealed by the BUSCO software. The obtained genomes were shorter than the previous assembly, GCA_000146385.1, which was 2,865,318 bp, excluding gaps between 83 contigs. The difference in the genome lengths could be due to selection for reduced genome size during the cultivation of the strain in laboratory conditions starting from the date of its isolation in 2010, or due to redundancy of multiple contigs of the previous assembly. The genomes obtained were annotated using the RAST Server and published at GenBank NCBI with the accession numbers CP033505.1 (assembly GCA_003827735.1) and CP033506.1 (assembly GCA_003827835.1) for the NC and FS variants, respectively. The staphylococcal methicillin-resistance chromosome cassette (SCC*mec*), comprising *mecA* and *mecR* genes (Holden et al., 2004; Jani et al., 2017), was found in both genomes ∼50 kbp downstream from the replication origin. However, the coverage of PacBio reads overlapping the SCC*mec* region decreased significantly in both NC and FS genomes, indicating that this mobile element could be unstable in the culture and that there was a tendency to drop this element when *S. aureus* was cultivated without the selective pressure of the antibiotic that is in agreement with previously published reports (Stojanov et al., 2015; Almebairik et al., 2020). This may explain the fact that both NC and FS cultures gained sensitivity to methicillin after 10 passages of cultivation on the media without the antibiotic (Table 1).

### Differential Gene Expression in *S. aureus* ATCC BAA-39 Variants NC and FS Under the Effect of FS-1

Gene expression patterns were identified in three repetitions by Ion Torrent sequencing of total RNA samples obtained from the NC and FS variants of *S. aureus* ATCC BAA-39. The scheme of the experiment is shown in Figure 1. Differential gene expression was analyzed by DESeq2 algorithm in 5 pairs of conditions: NC^–^/NC^+^, FS^–^/FS^+^, NC^+^/FS^+^, NC^–^/FS^+^ and NC^–^/FS^–^ denoted as *control/experimental* condition, where NC and FS are respective culture variants growing on the medium supplemented with FS-1 (+) or on the same medium without the drug FS-1 (−). Fold change and *p*-values of the differential gene expression in *S. aureus* ATCC BAA-39 at different growth conditions is shown in Supplementary Table 1.

**FIGURE 2:**
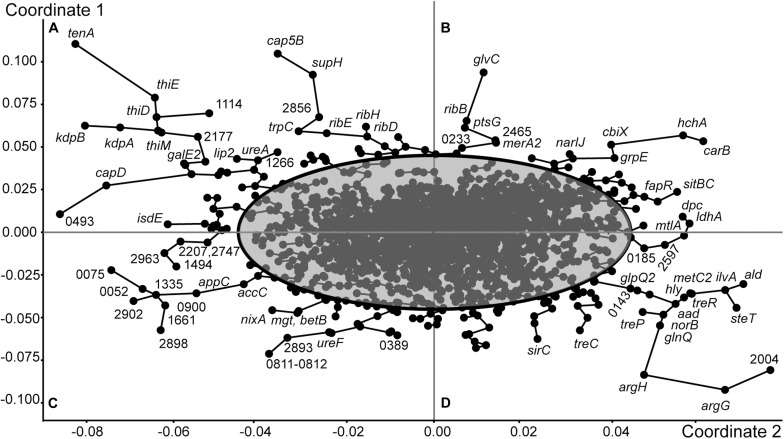
Grouping of genes of *S. aureus* ATCC BAA-39 by the Principle Coordinates Analysis (PCoA) algorithm based on their fold change expression data at 5 tested conditions: NC^–^/NC^+^, FS^–^/FS^+^, NC^+^/FS^+^, NC^–^/FS^+^ and NC^–^/FS^–^. Nodes representing individual genes are linked by minimal spanning tree graphs. 95% Confidence ellipse outlines the genes showing specific patterns of expression at different growth conditions. The outermost genes with condition-specific patterns of expression are labeled either by their gene names or by locus tag numbers as in the genome *S. aureus* ATCC BAA-39NC (CP033505.1). The figure is split by principle coordinates into four sectors, **(A)**, **(B**), **(C)** and **(D)**. Genes located in the same sector share their patterns of transcriptional regulation.

Grouping of genes of *S. aureus* ATCC BAA-39 by the Principle Coordinates Analysis (PCoA) algorithm based on their fold change expression data at 5 tested conditions is shown in Figure 2. Clusters of co-regulated genes are organized in tree branches linked by minimal span tree graphs and distributed between sectors A, B, C and D created by principle coordinates 1 and 2. The gene nodes lying outside of the 95% confidence ellipse are characterized by statistically reliable co-expression patterns. These genes show counter-regulation in opposite sectors. For example, the genes of sector A were characterized by differential levels of expression NC^–^ < FS^+^ < FS^–^ < NC^+^, while the expression levels of the genes of sector D was NC^+^ < FS^–^ < FS^+^ < NC^–^. These genes were strongly up- or down-regulated in the culture NC in response to the addition of FS-1 to the medium. In the culture FS adapted to the presence of FS-1 in the medium, these genes were continuedly expressed on an average level and did not significantly respond to the addition of FS-1. Unexpectedly, the expression of these genes in the culture FS was stronger when it was transferred to the medium without FS-1; however, the gene expression variations between the conditions FS^–^ and FS^+^ in many cases were statistically unreliable (*p*-values > 0.05). The most regulated genes of sector A were *tenA* aminopyrimidine aminohydrolase of the thiamine salvage pathway; genes of the thiamine biosynthetic pathway *thiDEM*; potassium-transporting ATPase *kdbAB*; extracellular polysaccharide biosynthetic genes *cap5B* and *supH*, and riboflavin biosynthetic genes *ribE*, *ribH* and *ribD*. The intensively regulated genes of sector D, which were strongly inhibited by FS-1, were L-arginine biosynthetic genes *argG* and *argH*; *ald2-ilvA1-steT-norB* operon associated with efflux pump synthesis and activation; lactose and maltose uptake and utilization genes *mtlA* and *ldhA*; alpha-phosphotrehalase *treC*; PTS threhalose transporter *treP* and trehalose operon transcriptional repressor *treR*.

The expression pattern of the genes of sector B is characterized by the following order of expression levels: FS^–^ < NC^–^ < NC^+^ < FS^+^; and the genes of sector C were regulated oppositely: FS^+^ < NC^+^ < NC^–^ < FS^–^. These genes were sensitized by 10 passages of cultivation with FS-1 and responded to the addition of FS-1 by a strong positive or negative regulation. In the culture NC, the differential expression of these genes at the conditions NC^–^ and NC^+^ in many cases was statistically unreliable. One of the most regulated genes of sector B is carbamoyl-phosphate synthase *carB*. Carbamoyl-phosphate synthesized by this enzyme may be further used either for biosynthesis of L-arginine or pyrimidines. Because the next steps of L-arginine biosynthesis by argininosuccinate synthase *argG* and argininosuccinate lyase *argH* were strongly downregulated at this condition, it may be assumed that the activation of *carB* was dictated by the need for DNA synthesis or repair. The activation of gene *carB* was co-regulated at these conditions with upregulation of protein and nucleotide deglycase *hchA*. Other co-regulated genes of sector B were genes for DNA lesion repair proteins (*uvrABC*); chaperon *dnaJ*; non-heme ferritin *ftnA*; spermidine/putrescine import protein *potA*; cobalamin biosynthesis ferrochelatase *cbiX*; respiratory nitrate reductase *narIJ*; iron/manganese ABC transporter *sitBC*; suppressor of fatty acid biosynthesis *fapR*; mercury reductase *merA2*; components of glucose and glucosamine PTS uptake systems *ptsG* and *glvC*; and ncRNA *teg25as* (HMPRNC0000_2674).

Oppositely regulated genes of sector C include biotin carboxylase *accC*; cytochrome *d* ubiquinol oxidase subunit *appC*; nickel/cobalt efflux protein *nixA*; peptidoglycan polymerase *mgt*; betaine biosynthetic gene *betB*; glycine/betaine transporter *opuD*; ribulosamine/erythrulosamine 3-kinase *yniA* involved in protein deglycation and 10 genes encoding regulatory ncRNA. Half of these ncRNA genes, HMPRNC0000_0900, 1335, 2206 (RNAIII), 2747 and 2902, were expressed only in the culture FS when it was cultivated on the normal medium without FS-1. Other five genes, HMPRNC0000_0052, 1456, 1494 (sRNA233), 1661 and 2390, were expressed also in the culture NC on the medium without FS-1. Addition of FS-1 to the medium completely abolished or significantly inhibited the expression of these regulatory elements. The presence of FS-1 in the medium halted the expression of three protein coding genes: ferrous iron transporter HMPREF0783_0807, periplasmic oligopeptide-binding protein *oppA* and ATP-binding subunit *clpL* of the Clp protease.

The effect of FS-1 on expression of several important genes involved in cell division and replication, and the genes of the methicillin resistance cassette (SCC*mec*) is shown in Table 2. As expected, the addition of FS-1 to the culture NC does inhibit the cell growth/division associated genes and antibiotic resistance genes. An unexpected observation was that the incubation of FS-1 adapted culture FS on the medium without the drug FS-1 caused an even stronger suppression of bacterial growth and the expression of the antibiotic resistance genes. Comparison of the gene expression in pairs of conditions, NC^–^/NC^+^ and FS^–^/FS^+^, confirmed that the removal of FS-1 from the medium stressed culture FS in a very much similar way as the addition of FS-1 to the medium stressed the culture NC. In total, 127 genes were regulated at both experimental conditions, NC^–^/NC^+^ and FS^–^/FS^+^, and 85 of these genes (67%) were counter-regulated meaning that the addition of FS-1 to the culture NC caused the same effect on these genes as the removal of FS-1 affected these genes in the culture FS. Such gene expression regulation may be associated with the response to the medium replacement stress. Remaining 42 genes, which were co-regulated at the experimental conditions NC^–^/NC^+^ and FS^–^/FS^+^, were of interest as they represented an effect caused by FS-1 independently whether the bacterial culture was adapted to the presence of the drug or not. There are 27 genes activated by FS-1 in both cultures, NC and FS, which include genes of Nar respiratory nitrate reductase operon; genes involved in biosynthesis of riboflavin (*rib*), molybdopterin (*moaC*), cobalamin (*cbiX*) and extracellular polysaccharides (*supH*); α-amylase *malA*; glucose and maltose uptake genes *ptsG*, *ptbA* and *glvC*; ribonucleoside diphosphate reductase subunits *nrdIE*; flavin-utilizing monooxygenase *yhbW*; oxidoreductase *nfrA*; thiol peroxidase *tpx*; mercuric reductase *merA2*; ribosomal protein S4 and SSU maturase *ylqF* important for translational accuracy and several other metabolic enzymes. There were 15 genes strongly inhibited by FS-1 in both cultures, NC and FS. They include *de novo* purine biosynthesis repressor *purR*; sugar phosphate antiporter *uhpT*; NADH-ubiquinone oxidoreductase *mnhA2*; alkaline shock gene *asp*; transmembrane transporter *gudP* and transporter of small amino acids and other nitrogen containing compounds *opuD*; sucrose uptake gene *scrA* and Clp protease gene involved in thermotolerance and intracellular multiplication.

**TABLE 2:** Altered expression of two groups of genes (i) involved in cell division, growth and replication; and (ii) genes of the methicillin-resistance chromosome cassette (SCC*mec*).

Locus tag	Gene name	Description	Fold change				
			NC^–^/NC^+^	FS^–^/FS^+^	NC^+^/FS^+^	NC^–^/FS^+^	NC^–^/FS^–^
**Cell division, growth and replication genes**							
0001	*dnaA*	replication initiator	−**2.3**	**4.79**	1.36	**−**1.69	−**7.89**
0002	*dnaN*	DNA polymerase III subunit	**−**1.25	**2.14**	**−**1.05	**−**1.3	−**2.73**
1690	*rpoD*	SigA primary sigma factor	**−**1.2	**3.1**	1.03	**−**1.17	−**3.53**
1691	*dnaG*	DNA primase	1.14	**19.2**	**−**1.01	1.11	−**16.7**
1818	*polA*	DNA polymerase I	**−**1.35	**5.24**	1.88	1.38	−**3.68**
1835	*dnaE*	DNA polymerase III subunit	1.06	**3.78**	1.03	1.09	−**3.36**
**Genes of the methicillin-resistance chromosome cassette** (SCC*mec*							
0042	*xylR*	Transcriptional regulator	−**17.2**	1.91	8.46	**−**1.92	**−**3.73
0043	*mecI*	Methicillin resistance repressor	**−**5.06	**−**1.91	4.66	**−**1.2	1.64
0044	*mecR*	Methicillin resistance regulatory protein	−**2.62**	2.97	**2.25**	**−**1.21	−**3.32**
0045	*mecA*	methicillin resistance determinant	**−**1.61	**4.89**	1.26	**−**1.29	−**6.02**
0046	*maoC*	Associated acyl dehydratase	**−**1.11	**−**1.06	**−**2.51	−**2.66**	**−**2.58

*Significant gene regulation with absolute fold change values equal or above 2.0 and statistical confidence p-values equal or below 0.05 are shown in bold.*

### Comparison of Gene Expression Patterns of FS-1 Treated *S. aureus* ATCC BAA-39 and *E. coli* ATCC BAA-196

**FIGURE 3:**
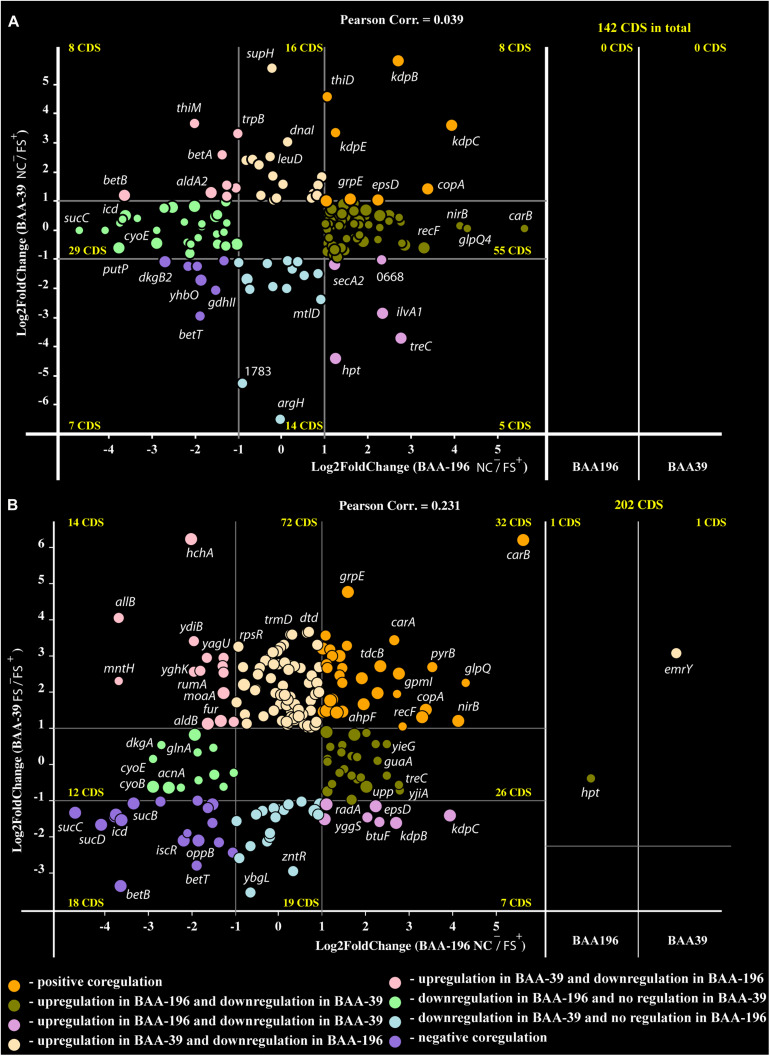
Co-regulation plots of fold-change values calculated for *E. coli* BAA-196 (X-axis) and *S. aureus* BAA-39 (Y-axis) at conditions **(A)** NC^–^/FS^+^ for both microorganisms and **(B)** NC^–^/FS^+^ for *E. coli* versus FS^–^/FS^+^ for *S. aureus*. Circles on the scheme colored according to the provided legend represent genes regulated in both genomes. Panels on the right parts of the plots represent those homologous genes, which were expressed at the given conditions only in one of the microorganisms, BAA-196 or BAA-39. The outermost regulated genes are labeled by their gene names.

Recently, an analysis of the effect of FS-1 on gene expression in *E. coli* ATCC BAA-196 at condition NC^–^/FS^+^ has been published (Korotetskiy et al., 2020b). In total, 725 homologous genes shared by the genomes *S. aureus* ATCC BAA-39 (CP033505.1) and *E. coli* ATCC BAA-196 (CP042865.1) were identified by the program GET_HOMOLOGUES. Comparison of fold change values calculated for homologous genes in *S. aureus* ATCC BAA-39 and *E. coli* ATCC BAA-196 under condition NC^–^/FS^+^ revealed only a few co-regulated or counter-regulated genes (Figure 3A). However, more significant positive correlation, 0.231, was obtained when the gene expression pattern in *E. coli* at condition NC^–^/FS^+^ was compared to the pattern of gene expression in *S. aureus* at condition FS^–^/FS^+^ (Figure 3B). Gene expression comparison at different conditions allowed the identification of commonly regulated genes in these distant model microorganisms under the effect of FS-1.

Only eight protein coding genes were positively regulated in FS^+^ compared to NC^–^ in both organisms (Figure 3A). These genes were heavy metal ion exporting P-type ATPase *copA* (Sitthisak et al., 2007), *kdpBC* K^+^-transporting ATPase subunits and DNA-binding response regulator *kdpE* expressed in bacteria in response to the osmotic stress (Epstein, 1986; Gründling, 2013); chaperons *dnaJ* and *dnaK*; capsular biosynthesis gene *epsD* and alkyl hydroperoxide reductase *ahpF* that protects cells against DNA damage by alkyl hydroperoxides. FS^–^/FS^+^ comparison confirmed that genes *copA* and *ahpF* are positively regulated by FS-1. Osmotic stress response genes *kdpBC* and *epsD* appeared counter-regulated at this condition as they were stronger activated in the *S. aureus* culture FS when it was transferred to the medium without the drug FS-1. It may be concluded that the presence of FS-1 in the medium and removal of this drug after culture adaptation to the presence of the drug, both invoke an osmotic stress to the bacterial culture. Genes *betAB* catalyzing biosynthesis of osmoprotectant glycine betaine from choline (Falkenberg and Strøm, 1990) were strongly downregulated by FS-1 in both microorganisms. The reason for this inhibition of betaine synthesis is unknown but may be associated with complexation of glycine-betaine with iodine (Blackwell et al., 2001).

**FIGURE 4:**
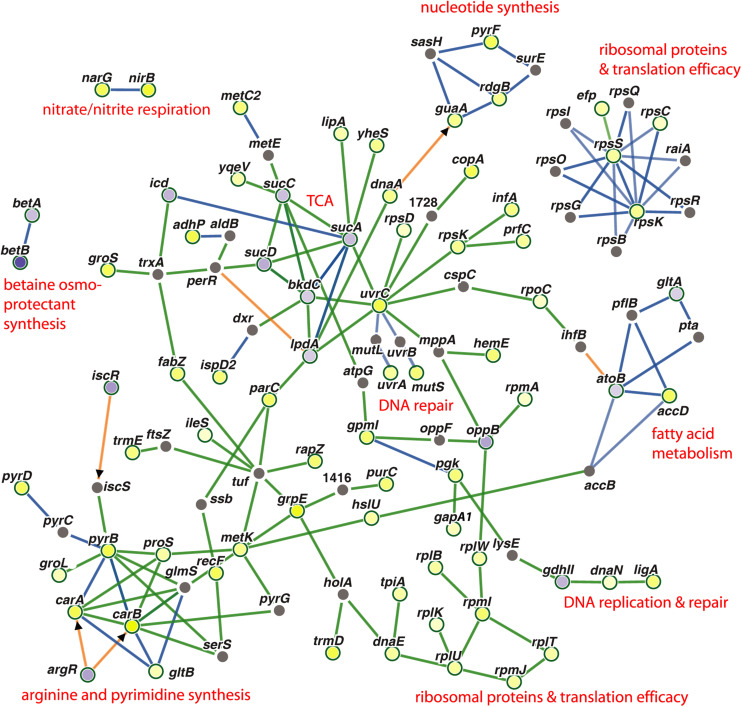
PheNetic networks of regulated genes grouped by functional or regulatory interaction between these genes or their products using the downstream regulation algorithm of the program PheNetic. Important functional groups of genes are labeled. Upregulated genes are depicted by yellow nodes and downregulated genes – by blue nodes (vertices). Color intensity indicates the level of transcriptional regulation (fold-change values). Gray nodes are genes or transcriptional regulators involved in the network, which expression was not reliably changed at the aforementioned conditions. Green edges show activation relations; blue edges – inhibitory relations; and brown edges – ambivalent or neutral relations. Direct regulation by transcriptional regulators are indicated by arrowheads.

Genes, which were co-regulated by FS-1 in *S. aureus* and *E. coli*, are summarized in Figure 4 by a PheNetic network of functionally associated genes. In both genomes, the presence of FS-1 in the medium had a strong inhibitory effect on enzymes of tricarboxylic acid cycle (TCA), *sucABCD*; fatty acids β-oxidation pathway (acetyl-CoA acetyltransferase *atoB*) and glyoxylate pathway associated with the fatty acid catabolism (citrate synthase *gltA*). In contrast, fatty acid biosynthesis was activated by upregulation of acetyl-CoA carboxyltransferase *accD*. The common response of *S. aureus* and *E. coli* to the presence of FS-1 in the medium was a switch to anaerobiosis that could be associated with the need to reduce the oxidative stress caused by iodine. Another hypothesis was that iodine may damage cytochrome molecules. Indeed, the transcription of all cytochrome *o* ubiquinol oxidase subunits, *cyoABCDE*, was brought to an almost complete stop in *E. coli*; however, in *S. aureus* these genes were constantly expressed. TCA cycle enzymes were strongly inhibited in *S. aureus* while fermentation genes, alpha-acetolactate decarboxylase *badA*, L-lactate dehydrogenase *ldhA*; formate acetyltransferase activating enzyme *pflA* and formate acetyltransferase *pflB*, were activated. This switch to the anaerobic lifestyle most likely was regulated by *arcR* that is the main regulator of anaerobiosis in *S. aureus* (Kohler et al., 2008). This transcriptional regulator was upregulated in NC and FS cultures when cultivated with FS-1. Fermentation related pathways were activated in *E. coli* by FS-1 in a similar way. Both these microorganisms used the nitrate/nitrite respiration pathway that was indicated by activation of nitrite reductase *nirB* and heme biosynthesis gene *hemE* (in both microorganisms), and the respiratory nitrate reduction chain, *narH*, *narJ*, *narH* and *narG*, in *S. aureus*. The analysis of the levels of expression of two NAD-dependent glyceraldehyde-3-phosphate dehydrogenases, *gapA1* (HMPRNC0000_0860) and *gapA2* (HMPRNC0000_1815), the former one is involved in glycolysis and the latter one controls gluconeogenesis in *S. aureus* (Chaffin et al., 2012), showed that the glycolysis was approximately 14-fold more active than the gluconeogenesis in *S. aureus* at the given conditions. Both these genes were upregulated in *S. aureus* FS that reflects a general activation of glycolysis and gluconeogenesis in the culture adapted to the presence of FS-1 in the medium. The transcriptional comparison at the conditions FS^–^/FS^+^ showed 5-fold upregulation of *gapA1* with no significant change in the level of expression of *gapA2*. Moreover, *cggR* repressor of gluconeogenesis (Kohler et al., 2008; Chaffin et al., 2012) was activated by FS-1. Such activation of glycolysis is typical for *S. aureus* growing at anaerobic conditions (Kohler et al., 2008); however, it was opposite to what was observed in *E. coli* where FS-1 treatment caused upregulation of the genes involved in gluconeogenesis and downregulation of their glycolytic counterparts (Korotetskiy et al., 2020b). It may be explained by the fact that *E. coli*, in contrast to *S. aureus*, has several alternative glycolytic pathways bypassing glycolysis, which are Entner Doudoroff, Embden–Meyerhof–Parnas and oxidative pentose phosphate pathways (Hollinshead et al., 2016). The genes of the Entner Doudoroff pathway, phosphogluconate dehydratase *edd* and keto-hydroxyglutarate-aldolase *eda*, were 1.8 and 1.6-fold upregulated in the FS-1 treated *E. coli* BAA-196.

Another commonality in the response of *S. aureus* and *E. coli* to FS-1 was activation of multiple enzymes involved in the nucleotide and protein biosynthesis, translational accuracy control and DNA repair. The latter group is represented by methyl-directed mismatch repair protein MutS, nucleotide excision repair proteins UvrA and UvrC, and NAD(+)-dependent DNA ligase LigA. Two chaperons, DnaJ and DnaK, also were activated in both microorganisms induced by FS-1.

There were only a few genes, which generally were counter-regulated by FS-1 in *S. aureus* and *E. coli* (Figure 3B). Two genes of the mixed acid fermentation pathway, fumarate reductase *frdA* and acetate kinase *ackA*, were strongly upregulated in *E. coli* but downregulated in *S. aureus*. Purine salvage hypoxanthine-guanine phosphoribosyltransferase *hpt* was expressed in *E. coli* and in the *S. aureus* culture NC, but it was transcriptionally silent in the *S. aureus* culture FS at all tested conditions. Moreover, this gene was 2.4-fold upregulated in *E. coli* at condition NC^–^/FS^+^, but in *S. aureus* this gene was 5-fold downregulated at condition NC^–^/NC^+^. This opposite response of one of the most important gene of purine turnover in *E. coli* and *S. aureus* remained unclear. Other oppositely regulated genes were cyclic pyranopterin phosphate synthase *moaA* involved in the molybdenum cofactor biosynthesis pathway, ferric uptake regulator *perR*, NADP-dependent acetaldehyde dehydrogenase *aldB* and carbonyl stress DNA and protein repair deglycase *hchA*. All these genes were strongly upregulated by FS-1 in *S. aureus* and downregulated in *E. coli*. The gene *lmrB2* encoding the multidrug efflux protein was transcribed only in the *S. aureus* culture NC and FS, but its homolog *emrY* in *E. coli* was transcriptionally silent at the given conditions. This gene was constantly expressed in *S. aureus* under condition NC^–^/FS^+^, but it was 3.3-fold upregulated under condition FS^–^/FS^+^.

In total, 91 genes were identified, which were differentially expressed under the effect of FS-1 only in *S. aureus* but showed no regulation by FS-1 in *E. coli*. Among specifically upregulated genes, there were several genes for ribosomal proteins (*rpmC*, *rpmD*, *rpsQ* and *rpsR*), DNA repair and carbon metabolism regulator *uvrY*, queuosine biosynthesis gene *queC*, arginine-tRNA ligase *argS*, D-aminoacyl-tRNA deacylase *dtd*, tRNA m^1^G37 methyltransferase *trmD*, chaperon *clpB* and chaperon associated protease *clpP*, purine biosynthesis gene *trxA* and shikimate 5-dehydrogenase *aroE*. Among genes showing a specific downregulation in *S. aureus* under condition FS^–^/FS^+^, there were zinc transport activator *zntR*, ATP synthase *atpA*, glycerol facilitator *glpF*, mixed acid fermentation fumarate reductase *frdB*, and lactam utilization 5-oxoprolinase *pxpA*.

### General Patterns of Modified Bases

Base modifications were predicted in three repeats of sequencing of DNA samples generated from NC and FS genomes. Epigenetically modified nucleotides, e.g., methylated, partially oxidized or halogenated residues, are characterized with significantly increased BM scores. However, BM scores of epigenetic modified nucleotides may be lower, if the modification occurred only in a fraction of the bacterial population.

**FIGURE 5:**
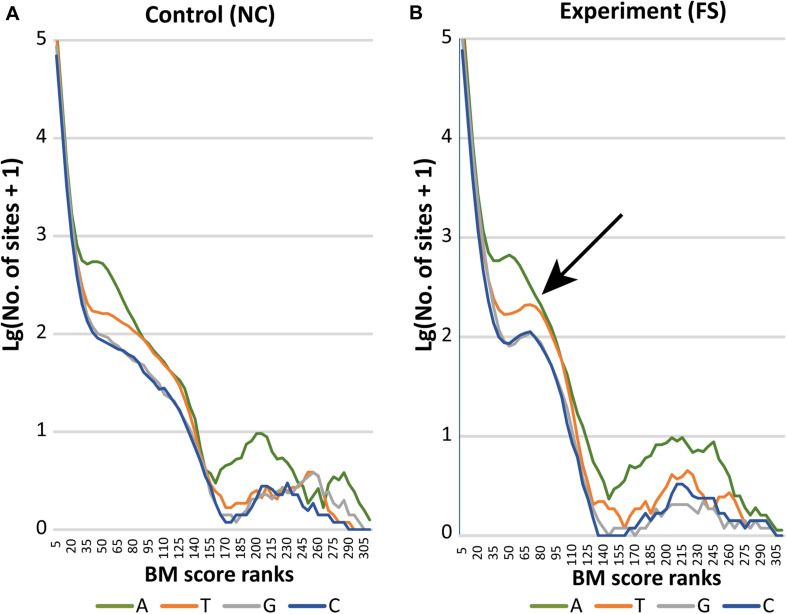
Distribution of BM scored nucleotides in the genomes **(A)** NC and **(B)** FS. Axis X presents BM score ranks stepping five score units. Axis Y indicates decimal logarithms of rank numbers. The specific increase of modified nucleotides in the genome of FS-1 treated *S. aureus* is pointed out by an arrow.

The distribution of BM scores calculated for all bases in NC and FS genomes of *S. aureus* is shown in Figure 5 by lines representing frequencies of adenine (A), thymine (T), guanine (G) and cytosine (C) residues along the BM score ranks. Axis Y represents decimal logarithms of the numbers of nucleotides per rank. In both genomes, 95% bases had BM scores below 14 and 99% bases had scores below 21. In the genome NC (Figure 5A), numbers of nucleotides with higher BM scores decreased gradually except for adenine residues having one peak in the range of BM scores from 50 to 65, and a larger escalation of adenine residues with BM scores 170–260. The latter peak corresponds to adenine residues N-methylated at sixth carbon atoms (m6A methylation). Similar increase in number of higher scored cytosine residues corresponds to methylation of these residues either at fourth or fifth carbon atoms (m4C and m5C methylation). Higher BM scores determined for guanine and thymine residues, but also for several adenine and cytosine residues, were associated with other types of epigenetic modifications; which cannot be recognized by the current version of the program *ipdSummary*. They were denoted as modA, modG, modT and modC.

In the genome FS, a drastic increase of all nucleotides with BM scores in the range from 50 to 95 was observed. In Figure 5B, these characteristic peaks are pointed out by an arrow. The area of escalation of high scored nucleotides (BM ranks 170–290) became wider in the genome FS.

### Patterns of Adenine Methylation in *S. aureus*

Adenine methylation and its role in gene expression regulation and adaptation of prokaryotes were discussed in many publications (Casadesús and Low, 2006; Bierne et al., 2012; Sánchez-Romero et al., 2015). Therefore, comparison of methylation patterns was in the focus of this study.

**FIGURE 6:**
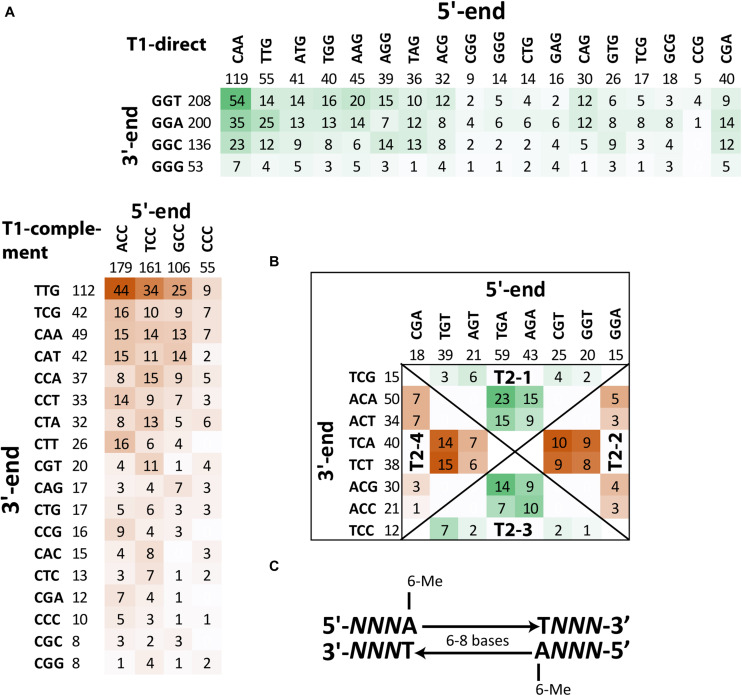
Frequencies of different BMF elements with m6A methylated sites in the genome *S. aureus* BAA-39 NC. **(A)** Frequencies of T1-direct/complement BMF elements. **(B)** Frequencies of T2-1/3-direct and T2-2/4-complement BMF elements. More frequent combinations of 5′-end and 3′-end semi-conserved triplets of BMF sequences are highlighted by color intensity. **(C)** Consensus sequence of BMF elements found in *S. aureus* BAA-39 NC.

Detailed analysis of context sequences adjacent to methylated adenine residues revealed that the majority of m6A sites were bipartite methylated fragments (BMF) of DNA with the first methylated adenine on the direct strand and the second one on the reverse complement strand with six or eight nucleotides in between (Figure 6C). According to REBASE classification (Roberts et al., 2015), this is type-I methylation that is often associated with DNA restriction-modification systems. Type-I methyltransferases are frequent in bacterial genomes and usually are comprised of three genes encoding a methyltransferase, motif recognition subunit and cognate restriction endonuclease. The best candidate for this role in the genome of *S. aureus* BAA-39 is type-I methyltransferase *hsdM* associated with the specificity unit gene HMPRNC0000_1947; however, there were no associated restriction enzymes around these genes. The addition of FS-1 to the medium has an opposite effect on the expression of these two genes (Supplementary Table 1) that implies their transcriptional control by different promoters. Comparison of the context sequences showed no significant sequence conservation of nucleotides enclosed by the bipartite m6A residues, except that they were AT-rich. Contrary, the triplets upstream and downstream of the m6A sites were semi-conserved (shown by capital *N*s in Figure 6C). The frequencies of combinations of different triplets at 5′- and 3′-ends of BMF elements are shown in Figures 6A,B. Two types of BMF elements were found. The sequences of type 1 were asymmetric (Figure 6A). On one side, the flanking triplet was NCC–m6A (or m6A–CCN in the reverse-complement variants), where N is A, T, G or C in order of the frequency of occurrence. The opposite flanking triplets were loosely conserved. They could be CAA, CGA, TTG, ATG, TGG, AGG, AAG, TAG, ACG, CAG, CAG, GTG, CTG, CTG, GAG, TCG, GCG, CGG, GGG, CGG, CCG, or CCG in order of the frequency of occurrence. Spacer sequences were 8 bp long. In total, there were 1,105 BMF elements of this type (82%). Pairs with loosely conserved sequences at the 5′-end and conserved 3′-end, NNN**A**-(n)_8_-*T*GGN, were denoted as direct type 1 BMF elements (T1-direct), as they were found predominantly in the leading replichore (the clockwise chromosomal sequence directed from the replication origin to the terminus). The most frequent motif of this type found on the chromosome 54 times was CAA**A**-(n)_8_-*T*GGT. The complement type 1 BMF elements, CCN**A**-(n)_8_-*T*NNN, were more frequent in the lagging replichore (counter-clockwise sequence from the replication origin to terminus) and thus denoted as T1-complement BMF elements (Figure 6A). The most frequent motif of this type found on the chromosome 44 times was ACC**A**-(n)_8_-*T*TTG. In the BMF motifs shown above, nucleotides in bold are methylated sites, and the nucleotides opposing the bipartite methylation on the complement DNA strand are denoted with an italic font.

**FIGURE 7:**
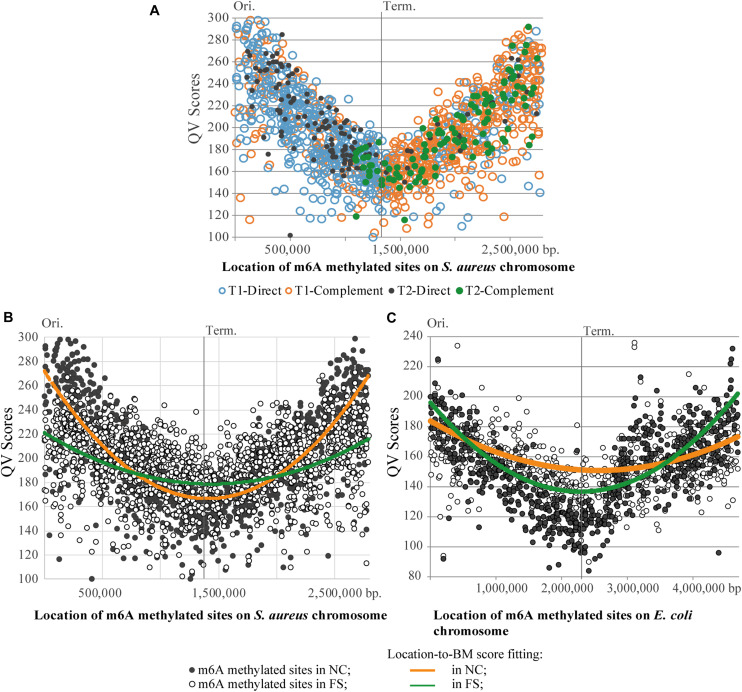
Distribution of m6A methylated sites on the chromosomes of *S. aureus* and *E. coli*. **(A)** Asymmetric distribution of the direct and complement variants of BMF elements on the leading and lagging chromosomal replichores of the *S. aureus* BAA-39 chromosome. **(B)** Degeneration of the parabolic QV score-to-location fitting of m6A methylated sites in the *S. aureus* BAA-39 FS chromosome compared to NC. **(C)** Degeneration of the parabolic QV score-to-location fitting of m6A methylated sites in the *E. coli* BAA-196 FS chromosome compared to NC. Calculated parabolic fitting lines of average BM scores are shown.

BMF elements of type 2 (they also may be classified as REBASE type-I methylation) have equally conserved flanking triplets at the 5′- and 3′-ends. These elements were shorter with 6 bp in the spacer regions. Four groups of these sequences were found. In Figure 6B, these groups are separated by diagonal lines and titled T2-1, -2, -3 and -4. Sequences of the groups T2-1 and T2-4 were defined as direct sequences (T2-direct) and sequences of the groups T2-2 and T2-3 were defined as complement sequences (T2-complement). Direct sequences were more frequent in the leading replichore and complement ones were frequent in the lagging replichore. In total, there were 240 instances of type 2 BMF elements (18% of all instances). The most frequent motif of the direct type is TGA**A**-(n)_6_-*T*ACA (23 instances), and TGT**A**-(n)_6_-*T*TCT (15 instances) is the most common motif of the complement type. The disproportional density of the direct and complement BMF elements found on the leading and lagging replichores are shown in Table 3 and illustrated in Figure 7A. This methylation should be controlled by an alternative methyltransferase. The most likely candidate is the orphan (not associated with any restriction enzymes) adenine-specific methyltransferase HMPRNC0000_1848 (upregulated in the medium with FS-1).

**TABLE 3:** Distribution of the direct and complement type 1 and 2 BMF elements on the chromosomal replichores.

Sequence type		Leading replichore	Lagging replichore
Type 1	Direct	420	176
	Complement	172	337
Type 2	Direct	89	40
	Complement	41	70

A common property of BMF elements of both types, direct and complement, is that the upstream m6A residues are almost always located on the direct DNA strand (clockwise from the replication origin), and the downstream counterpart m6A residues are on the reverse complement strand. Of 1,355 BMF elements found in NC and FS genomes, only two in NC and four in FS were in reversed order, with the upstream m6A on the reverse-complement strand. All these facts demonstrate that the location and orientation of BMF elements on the chromosome of *S. aureus* is of importance for this microorganism.

The total number of BMF elements in NC and FS genomes remained the same: 1,355. The complete list of identified BMF elements with respective locations, coverage and BM scores is in Supplementary Table 2. Among them, 1,321 BMF elements were at the same locations on the chromosomes NC and FS, but in 34 cases the BMF elements in NC were replaced with alternative elements in FS. Ten such replacements were in the body of the methicillin resistance cassette SCC*mec*. BMF were infrequent in SCC*mec* compared to other parts of the chromosome including identified prophages. It may be concluded that the SCC*mec* insert was subjected to alternative methylation depending on the growth condition and stresses experienced by the microorganism.

A striking difference in profiles of the m6A methylated sites in the NC and FS variants of *S. aureus* was in the distribution of BM scores of the m6A nucleotides along the chromosome (Figure 7B). In NC, the BM scores were significantly higher near the chromosomal origin of replication, but they decreased progressively in the direction toward the replication terminus on both replichores. The culture cultivated with FS-1 showed a different distribution of BM scores along the chromosome with a smaller difference between the BM values of the m6A sites around the replication origin and the terminus. BM scores indicate either the efficacy of methylation of the site (proportion of methylated nucleotides at this location in the population) or the local coverage. Analysis of coverage values of PacBio reads aligned against the genome consensus sequences showed the same parabolic distribution with lower values around the replication terminus (Supplementary Figure 1). Due to the continuous replication, the areas around the replication origin in bacterial cells are semi-diploid that increases the coverage of randomly generated reads compared to the area around the replication terminus (Marczynski and Shapiro, 1993). It may be assumed that in bacterial populations with different distribution of dividing and stationary cells, the shape of the coverage curve will be different. The treatment of *S. aureus* with FS-1 caused an obvious phase shift in the population illustrated in Figure 7B by the average BM score lines. Strangely, the treatment of *E. coli* BAA-196 cells with FS-1 affected the population oppositely by making the coverage/BM score curves more concave in the FS-1 treated culture (Figure 7C). The chromosome of the latter organism also was rich with m6A methylated sites; however, the major methylation motif was typical for Enterobacteriaceae REBASE type-II Dam methylation GA*T*C (Marinus and Løbner-Olesen, 2014; Blow et al., 2016). Only a small fraction of bipartite m6A methylated sites were associated with type-I methylation motifs AAC-(n)_6_-G*T*GC and GCAC-(n)_6_-G*T*T, which resemble the corresponding BMF elements of the genome of *S. aureus* BAA-39.

### Patterns of Other Epigenetically Modified Nucleotides

Methylated cytosine residues identified by the program *ipdSummary* as m4C methylated sited were infrequent and they were not associated with any sequence motifs. However, several m4C sites showed high BM scores in all three PacBio SMRT sequencing repeats. In contrast to the m6A methylated sites discussed above with preserved locations in both the genomes, the m4C methylated nucleotides, even those with the highest BM scores, were distributed differently in the genomes NC and FS. Table 4 shows the distribution of the high scored m4C sites with BM scores above 160. This cutoff value was selected by analyzing the BM score distribution shown in Figure 5. Only 5 high scored m4C sites out of 15 in NC and 19 in FS genomes were located at the same positions in both genomes: two sites in the genes hyaluronate lyase precursor *hysA* and DNA-methyltransferase *hsdM*, and other three sites in non-coding regions. Other genome specific high scored m4C sites were found in several coding sequences and frequently in transcribed regions not associated with protein coding genes, which could be unidentified genes for regulatory RNA molecules. It may be assumed that the alternative m4C methylation potentially can interfere with these regulatory elements. Two FS specific high scored m4C sites were found in the methicillin resistance cassette SCC*mec*. No cytosine specific methyltransferases were found in the genome of *S. aureus* BAA-39 except for two uncharacterized orphan methyltransferases HMPRNC0000_1724 and HMPRNC0000_1746 (both are significantly down-regulated at the FS^–^ condition).

**TABLE 4:** High scored cytosine methylated (m4C) sites identified in the genome NC and FS.

Genome NC						Genome FS					
N	Location	BM score	Context*	Strand	Annotation	N	Location	BM score	Context*	Strand	Annotation
1,2	69275- 69283	184	AT**C**TAT**C**GC	rev	transcribed non-coding sequence	1	10267-10275	244	GATA**C**GTAT	rev	fragmented ADP-dependent (S)-NAD(P)H-hydrate dehydratase
3	106655-106663	181	G**A**GT**C**G*T*GT	dir	non-coding	2	50038-50046	180	AAAG**C**TGCG	rev	transcribed hypothetical protein within methicillin resistance cassette SCC*mec*
4,5	198408-198418	223	AG**C**TGTAC*G*AT	dir	transcribed non-coding sequence	3	50829-50837	201	ACAA**C**GTAT	rev	transcribed non-coding sequence within methicillin resistance cassette SCC*mec*
6	303122-303130	162	TTTT**C**AATA	dir	non-coding	5	615296-615304	169	ACATCGCAT	dir	non-coding
7	334792-334800	206	CAAG**C**ATGG	dir	transcribed non-coding sequence	6	758206-758214	184	ATAA**C**GTGT	dir	non-coding strand of 7-carboxy-7-deazaguanine synthase *queE*
8	368158-368166	183	GTAT**C**GTAT	rev	Promoter of glycerol-3-phosphate transporter *glpT*	7	1147316-1147324	224	TCTG**C**GCAT	dir	non-coding
9	585346-585354	161	CGTG**C**GGGG	rev	transcribed non-coding sequence	8	1572394-1572402	199	CAAA**C**GTAA	dir	non-coding
10	1825322-1825330	174	AAGT**C**TAGT	dir	transcribed non-coding sequence	9,10	1789630-1789639	163	G**A**TG**C***G*CAAT	rev	transcribed non-coding sequence
						11	1798863-1798871	170	GCTA**C**GATA	dir	transcribed non-coding sequence
**11^†^**	1864270-1864278	186	TACT**C**AAGG	dir	Hyaluronate lyase precursor *hysA*	**12**	1866603-1866611	164	TACT**C**AAGG	dir	Hyaluronate lyase precursor *hysA*
**12**	1872123-1872131	166	GCGA**C**GGCG	rev	DNA-methyltransferase *hsdM*	**13**	1874453-1874461	172	GCGA**C**GGCG	rev	DNA-methyltransferase *hsdM*
						14	1915302-1915310	163	AAGA**C**AGAT	rev	non-coding
						15	1915353-1915361	182	AATT**C**GCAT	dir	non-coding
						16	1918103-1918111	181	CGCT**C**TTAC	dir	transcribed non-coding sequence
**13**	1961139-1961147	169	GGGC**C**CCTG	dir	non-coding	**17**	1963159-1963167	177	GGGC**C**CCTG	dir	non-coding
**14**	2090832-2090840	222	ATTT**C**ATTT	rev	non-coding	**18**	2092792-2092800	219	ATTT**C**ATTT	rev	non-coding
**15**	2584626-2584634	170	GGGC**C**CCTG	dir	non-coding	**19**	2586419-2586427	164	GGGC**C**CCTG	dir	non-coding

*^∗^In the context sequences, nucleotides shown in bold are methylated, and the nucleotides opposing the bipartite methylation on the complement DNA strand are denoted by italic typeface. ^†^Methylation sites located in the same regions in both genomes are marked by ordinal numbers shown in bold.*

**FIGURE 8:**
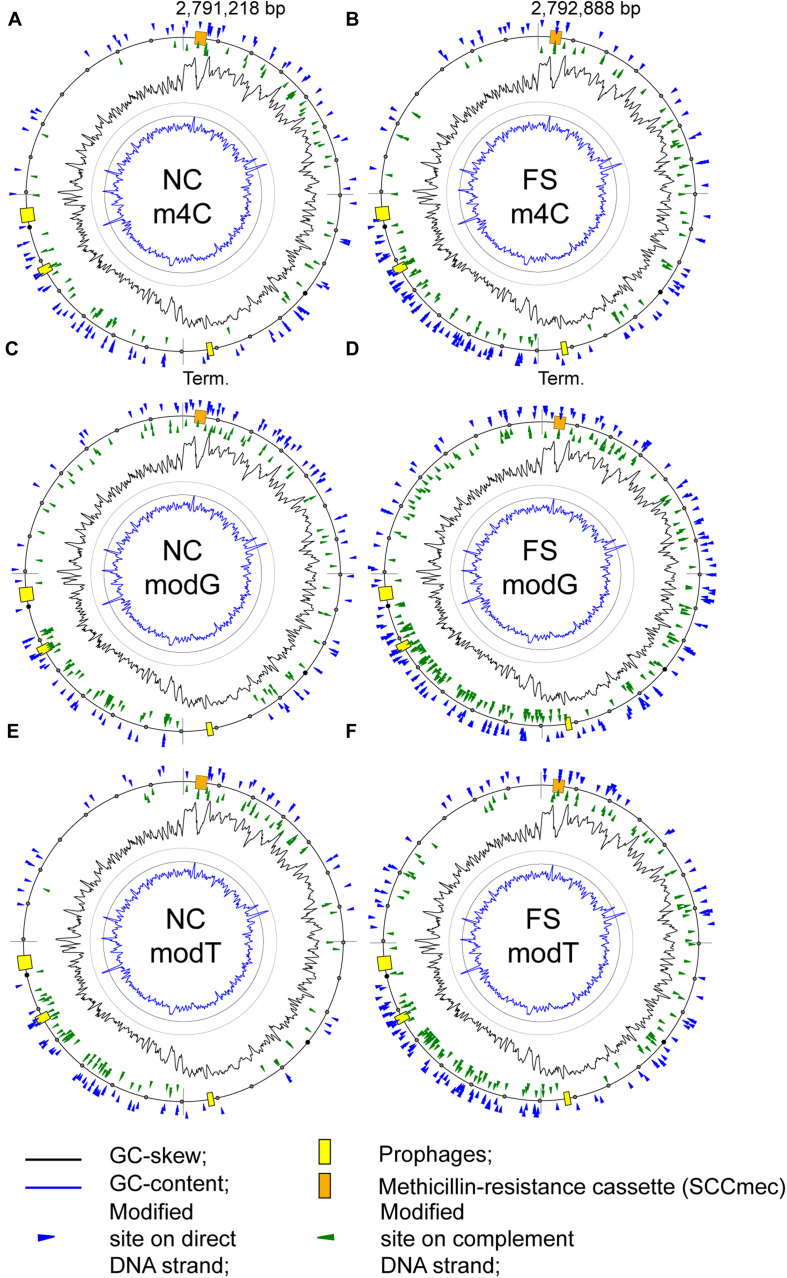
Distribution of modified nucleotides with BM scores above 70 on the chromosomes of the *S. aureus* BA-39 NC and FS variants. **(A)** m4C methylated sites in NC; **(B)** m4C methylated sites in FS; **(C)** modG sites in NC; **(D)** modG sites in FS; **(E)** modT sites in NC; and **(F)** modT sites in FS.

There was an increase of modified nucleotides of all types with moderate BM scores in the range from 70 to 95 in the FS-1 treated *S. aureus* (Figure 5). The distribution of these modified nucleotides was further analyzed as shown in Figure 8. An increased density of modified nucleotides was observed in the second half of the lagging replichore (downstream of the replication terminus) and in the first half of the leading replichore (downstream of the replication origin). This disproportional distribution of the modified bases was observed in both genomes; however, there are more modified bases in the genome FS compared to NC.

A possible effect of base modifications on the gene expression level was analyzed. Taking the average level of gene expression in the culture FS as 1, the level of expression of genes with additional modG modifications compared to NC was 0.732 on average, while in those genes with unchanged number of modG, the expression level was 1.134 on average. There was a clear tendency of avoidance or counter-selection of modG modifications from highly expressed genes. However, it should be admitted that because of the significant variability in gene expression values within the groups, the difference between average values was statistically unreliable, meaning that there were many other factors affecting gene expression besides the modG modifications. When the numbers of modA, modG, motT and modC modifications were considered, the difference in the level of gene expression was smoothed out to 0.836 for the genes with novel nucleotide modifications and 1.096 for the unmodified genes. It shows that the other types of modifications did not contribute to the effect of avoidance of highly expressed genes, or their effects were ambivalent.

### Complexing of FS-1 With Bacterial DNA

Epigenetic changes in bacterial chromosomes under the effect of the iodine-containing nano-molecular complex FS-1 suggested possible complexing of DNA with nano-micelles and/or with the released iodine, which can potentially halogenate chromosomal nucleotides, predominantly thymine residues (Ilin et al., 2013). To check this hypothesis, complexing of FS-1 with the chromosomal DNA was investigated experimentally.

To study the ability of FS-1 to penetrate bacterial cell wall and create complexes with the chromosomal DNA, the drug was synthesized with the radioactive isotope ^131^I (20 MBq/ml). The residual radioactivity was measured in DNA samples extracted from the treated-and-washed cells. It was found in three repetitions that the radioactivity of the extracted DNA constituted 12.76% ± 9.04 of the residual radioactivity of the treated cells that corresponded to 0.14 ± 0.015 Bq/ng of the extracted DNA. The residual radioactivity can be associated with both complexing of FS-1 with DNA and with a direct halogenation of nucleotides by iodine isotopes. In a similar study with *E. coli* ATCC BAA-196, the residual radioactivity of the extracted DNA constituted only 0.46% ± 0.15 of the residual radioactivity of the treated bacterial cells measured after washing the cells (Korotetskiy et al., 2020b).

## Discussion

Drug-induced reversion of antibiotic-resistant bacterial pathogens into antibiotic-susceptible phenotypes is a promising way to control outbreaks of drug-resistant infections. There are two basic approaches to antibiotic resistance reversion: (i) direct inhibition of the resistance mechanisms; and (ii) active selection against drug resistance in bacterial populations using evolutionary and physiological interactions between drugs and bacteria. The latter approach was discussed and modeled in the review by Baym et al. (2016); however, these authors did not gain deep insight into possible molecular mechanisms of counter-selecting against drug resistance in bacterial populations because of absence of workable models. The introduction of FS-1 into clinical practice (Ilin and Kulmanov, 2014; Ilin et al., 2017; Korotetskiy et al., 2017; Islamov et al., 2018; van Niekerk et al., 2018) allowed detail investigation of this phenomenon. A series of experiments on selected multidrug resistant model microorganisms was performed, first on *E. coli* ATCC BAA-196 (Korotetskiy et al., 2020b) and currently on *S. aureus* ATCC BAA-39. FS-1 steadily converts the treated bacteria to antibiotic-sensitive phenotype. Bacteria remain sensitive even after the removal of FS-1 from the medium at least for several generations. While the FS-1 treated bacterial populations regain the antibiotic resistance at *in vitro* experimental conditions under the selective pressure of antibiotics (data on the dynamics of antibiotic resistance regaining were not shown due to significant variations between the repeated experiments), the *in vivo* experiment published before showed a promising therapeutic effect of co-administration of FS-1 with antibiotics (Ilin et al., 2017). This drug can be applied prior to antibiotics or in combination with them. Supplementary drug may induce phase variations in bacterial populations and favor the selection of drug-sensitive variants. Mechanisms of phase variations can be genetic, such as mutations in genes and promoter regions (Henderson et al., 1999; Gründling, 2013), or epigenetic, including altering methylation/modification patterns (Casadesús and Low, 2006; Low and Casadesús, 2008; Phillips et al., 2019a), nucleotide oxidation and DNA phosphorothioate modifications (Liu et al., 2019). Recent publications demonstrated the important role of phase variations in host-pathogen and host-environment interactions, and in bacterial adaptation (Haagmans and van der Woude, 2000; Hernday et al., 2002; van der Woude and Bäumler, 2004; Phillips et al., 2019a,b). The role of epigenetic modifications in antibiotic resistance development has also been demonstrated (Nyce et al., 1993; Adam et al., 2008). Nevertheless, the possibility to use induced phase variations to revert antibiotic resistance has never been explored. In this work, a third-generation sequencing approach was used to investigate possible genetic and epigenetic mechanisms of antibiotic resistance reversion induced by FS-1 in *S. aureus* ATCC BAA-39.

In the conducted experiment, *S. aureus* culture was cultivated in test-tubes for 10 days with daily re-inoculations to fresh MH medium containing 1/2 MBC FS-1 (450 μg/ml). This culture was denoted FS. Negative control (NC) culture was cultivated in parallel on the MH medium without FS-1.

An expectation was that antibiotic resistance reversion may be induced by altering DNA methylation profiles. Methyltransferases identified in the genome of *S. aureus* BAA-39 were not associated with DNA restriction-modification systems and played other roles rather than the protection of DNA against cognate restriction enzymes. It was hypothesized that type-I methylation in this genome may be associated with architecture-imparting sequences (AIMS). The m6A methylation pattern of *S. aureus* BAA-39 showed well-organized global replichor-oriented distribution of BMF elements that resembles the distribution of AIMS on bacterial chromosomes (Hendrickson et al., 2018). AIMS are semi-conserved 6-8 bp sequences preferentially abundant on the leading strand. AIMS may be synonyms of FtsK orienting polar sequences (KOPS) assisting the directional loading of the FtsK translocases (Bigot et al., 2006), which are important for proper chromosomal replication and DNA repair. Spacer regions of BMF elements in *S. aureus*, as well as in *E. coli*, were 6-8 bp long, the same as AIMS. Further study should be performed to investigate possible relationships between AIMS, KOPS and BMF elements as architectural units of bacterial chromosomes. The absence of AIMS in horizontally acquired genomic islands suggests a possibility that these sequences may help to prevent insertion of mobile genetic elements by allowing a higher frequency of mutations in these inserts (Hendrickson et al., 2018). The global m6A methylation pattern persisted the treatment with FS-1 except for the SCC*mec* genomic island. The treatment with FS-1 increased instability of this regions and possibly promoted its excision from the chromosome of *S. aureus* due to improper methylation of internal BMF elements. Indeed, a drop of coverage of PacBio DNA reads generated from this region was observed in both, NC and FS cultures, suggesting an excision of the genomic islands at least in a fraction of cells; however, the coverage difference between NC and FS genomes was insignificant. A similar destabilization of the virulence plasmid in the FS-1 treated *E. coli* BAA-196 was reported in a previous publication (Korotetskiy et al., 2020b). The varying rate of excision of SCC*mec* cassettes from Staphylococcal populations was reported in another publication (Almebairik et al., 2020). However, it should be noted that in the current work, the rate of SCC*mec* excision in NC and FS cultures was not measured. The hypothesis of destabilization of antibiotic resistance genetic inserts by the treatment with FS-1 must be checked in future studies.

The altered adenine and cytosine methylation landscape in the FS-1 treated *S. aureus* was clearly demonstrated; however, it should be accepted that the role of the differential nucleotide methylation must be proved in further studies as the altered patterns of methylation equally likely may be either gene regulation factors or the result of differential gene regulation at different growth conditions. Nevertheless, the repeated culture-specific methylation of the NC and FS genomes of *S. aureus* confirmed in three repetitions of PacBio genome sequencing demonstrates a biological importance of this phenomenon and possibility to use the methylation landscape as a genetic marker of the initial and FS-1 treated culture variants.

**FIGURE 9:**
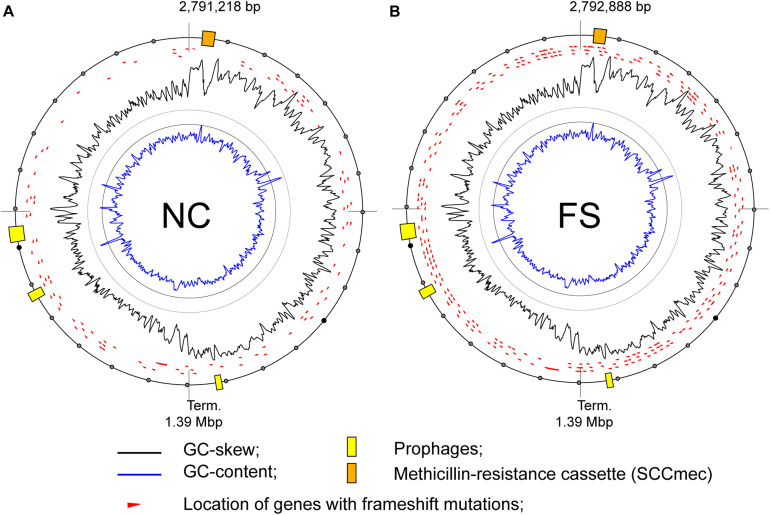
Locations of genes with frame-shift mutations on the chromosomes of the *S. aureus* BA-39 variants **(A)** NC and **(B)** FS.

The treatment of bacterial cultures with FS-1 caused an increase in numbers of modified nucleotides in the chromosome of *S. aureus* (Figures 5, 8). These global epigenetic modifications, which chemical nature remained disputable, could be an integral part of induction of antibiotic resistance reversion. Relatively low BM scores (65–80) of identified modified sites and the absence of contextual motifs imply a random pattern of nucleotide modifications caused by FS-1. Analysis of the distribution of modG sites showed a tendency to avoid highly expressed genes that may indirectly indicate an interference of the modG modifications with the gene transcription or an increased rate of mutation of the affected genes. The modG modifications may correspond to O-6-guanine methylation resulted from an abnormal activity of DNA methylases. O-6-methylguanine is a highly mutagenic adduct provoking single nucleotide mismatches during replication (Gu et al., 2017). Guanine residues may be oxidized to 7,8-dihydro-8-oxoguanine that is another strong mutagen due to its complementation with both cytosine and adenine (Neeley and Essigmann, 2006). It may be expected that the rate of mutations should increase in the FS-1 treated *S. aureus*. Frequent re-inoculations and the growth on the simple nutrient rich medium were favorable for accumulation of genetic mutations in genes, which may be out of importance at this laboratory condition. In total, 104 protein coding genes in NC and 271 genes in FS were truncated owing to frameshift mutations. The distribution of truncated genes in both genomes identified by comparison to the initial whole genome sequence of this microorganism (AEEK00000000.1, published in 2013), is shown in Figure 9. Particularly, gene *mecA* encoding for an alternative penicillin-binding methicillin-resistant protein PBP2a has a frameshift mutation at its 5′-end in the genome FS that makes the encoded protein shorter by 61 amino acids. Alignment of Ion Torrent reads generated from the total RNA samples showed that the mutated gene was transcribed possibly from an alternative promoter. The level of expression of this gene was six-fold downregulated in FS compared to NC grown on the medium without the drug FS-1. It may be a result of either this mutation, or an alternative gene regulation, or excision of the genomic island from the chromosome. The neighbor gene *mecR* encoding for methicillin resistance regulatory sensor-transducer was 3-fold down-regulated in FS at this condition (Table 2).

The gene expression patterns in *S. aureus* and *E. coli* induced by the treatment with FS-1 showed some level of similarity (Figure 3). While the calculated Pearson correlation coefficient, 0.231, is moderate, it could not be higher as the homologous genes of these phylogenetically distant microorganisms were expressed differently even at the same condition (data not shown). An interesting finding was that the general pattern of gene expression in the negative control *E. coli* was more similar to that in the FS-1 treated *S. aureus* rather than to NC culture (Figure 3). Nevertheless, there was a commonality in the general trend of gene regulation in both microorganisms (Figure 4): strong down-regulation of the TCA pathway; switch from aerobic respiration to anaerobic respiration or fermentation; increased fatty acid synthesis with prohibited β-oxidation of fatty acids; activated synthesis of proteins and nucleotides; activation of genes associated with osmotic stress response but strong downregulation of synthesis of betaine osmoprotectants; and selective activation or inhibition of transmembrane transporters possibly caused by an attempt to diminish the uptake of iodine-containing compounds. Noteworthily, while *S. aureus* may survive anaerobic or microaerophilic conditions for long time, anaerobiosis affects strictly the virulence and antibiotic resistance potential of this bacterium (Fuchs et al., 2007). The transition to anaerobiosis caused by the treatment with FS-1 may explain the observed increase in susceptibility to antibiotics (Table 1).

One striking difference was the strong upregulation of nucleotide deglycase *hchA* in the FS-1 treated *S. aureus*. HchA is a component of the major nucleotide glycation repair system activated under carbonyl stress that reverses methylglyoxal and glyoxal damage mostly in guanine residues via nucleotide sanitization and direct nucleic acid repair (Richarme et al., 2017). Carbonyl stress in bacteria is associated with strong oxidative stress and intracellular accumulation of various reactive carbonyl compounds, which may be halogens and their products (Semchyshyn and Lushchak, 2012; Kosmachevskaya et al., 2015; Ezraty et al., 2017). Nitrogen and oxygen atoms in purine and pyrimidine bases are the most attractive targets for reactive carbonyl compounds, which lead to their chemical modification resulting in cytotoxicity and mutagenicity (Ellis, 2007; Liu et al., 2010; Semchyshyn and Lushchak, 2012). In *E. coli* treated with FS-1, the homologous gene *hchA* was downregulated. It suggests that the transmembrane flux of iodine-bound compounds into FS-1 treated *E. coli* cells was significantly lower even that in both cases FS-1 was applied in 1/2 MBC concentrations calculated for respective microorganisms (450 μg/ml for *S. aureus* and 500 μg/ml for *E. coli*). In contrast to *S. aureus*, no increase in the number of modified nucleotides and no increase in the rate of frameshift mutations was observed in *E. coli* treated with FS-1 (Korotetskiy et al., 2020b). In concordance with this was the detection of only traces of the radioactive iodine in the DNA extracted from the cells of *E. coli* treated with ^131^I marked FS-1, while in the respective DNA sample extracted from the treated *S. aureus* cells, the residual radioactivity of DNA constituted 12.76% ± 9.04 of the total radioactivity of washed bacterial cells. One commonality in the response of *S. aureus* and *E. coli* to the treatment with FS-1 in terms of epigenetic modifications lies in an alternative distribution of high scored methylated cytosine residues that could be a common mechanism of population phase variation in response to environmental stresses.

A recent study confirmed an activation of genes associated with the oxidative stress response in *S. aureus* in 5 min after injection of FS-1 into growth medium (Korotetskiy et al., 2017). Changing of the redox potential in proximity with the chromosomal DNA may promote the oxidation of nucleotides. Moreover, close co-location of two oxidized guanine residues on the chromosome can lead to double-strand DNA breaks and cell death (Foti et al., 2012). These authors claimed also that closely spaced 8-oxo-deoxyguanine lesions leading to lethal double-strand DNA breaks and RNA mistranslation contribute significantly to the cytotoxicity of many broadly used antibiotics including beta-lactams, quinolones and aminoglycosides. In this case, the DNA oxidation by FS-1, which may be inherited by bacterial daughter cells in several generations, may increase the sensitivity to antibiotics even when FS-1 is removed from the medium.

## Conclusion

This study demonstrated reversion of antibiotic resistance obtained on the reference multidrug resistant strain *S. aureus* ATCC BAA-39 treated with the iodine-containing nano-micelle drug FS-1. Comparison of complete genome sequences, transcriptional profiles and landscapes of epigenetic modifications suggested several possible molecular mechanisms of the antibiotic resistance reversion, which include (i) profound transition of bacterial metabolism toward anaerobiosis due to damaging of oxygen-depended terminal cytochrome:ubiquinol electron transfer complexes by iodine that eventually caused unfavorable conditions for bacteria to maintain the initial level of antibiotic resistance; (ii) increased rate of mutations in the FS-1 treated cells due to oxidation and/or halogenation of chromosomal DNA nucleotides that eventually may affect many important cellular systems including antibiotic resistance genes; (iii) osmotic, oxidative and carbonyl stresses experienced by the FS-1 treated cells may aggravate the antibiotic resistance fitness cost; (iv) the treatment with FS-1 may cause destabilization and loss of horizontally acquired antibiotic resistance genomic islands (e.g., SCC*mec*) and virulence plasmids that was reported for the FS-1 treated *E. coli* BAA-196 (Korotetskiy et al., 2020b).

In total, the expression of 2,952 protein or regulatory RNA coding genes of *S. aureus* BAA-39 participating basically in all biological processes were affected during 10 passages of the cultivation with FS-1 (Supplementary Table S1). Further study will aim at identification and experimental validation of common mechanisms of action of iodine-containing nano-micelles on taxonomically distant microorganisms, *S. aureus*, *E. coli* (Korotetskiy et al., 2019a, 2020b), *A. baumannii* (Korotetskiy et al., 2020a) and *M. tuberculosis* (Ilin et al., 2015, 2017). The drug FS-1 has successful passed clinical trials in Kazakhstan as a supplement of the antibiotic therapy against multidrug resistant tuberculosis^4^ (acc. NCT02607449). Future studies will identify specific aspects of the action of the drug on other multidrug resistant pathogens causing nosocomial infection outbreaks to allow better formulation of novel drugs against each individual pathogen. The comparison of the gene expression patterns of *S. aureus* and *E. coli* carried out in this work has showed several important particularities of the responses of these bacteria to FS-1. While *S. aureus* suffered mostly from DNA damaging, oxidative and carbonyl stresses, *E. coli* seemed to be more protected from the influxes of iodine into the cells possibly due to much stronger inhibition of almost all transmembrane nutrient uptake system and cytochrome:ubiquinol electron transfer complexes. However, this strategy led the FS-1 treated *E. coli* culture to a nutrient stringency and stronger osmotic stress. Further studies will be performed to ensure that these responses including the antibiotic resistance reversion are inherent also in clinical isolates of nosocomial infections. This work will be performed on a collection of multidrug resistant strains isolated recently from clinics in Kazakhstan, sequenced and deposited at NCBI (Korotetskiy et al., 2019b).

## Data Availability Statement

The datasets presented in this study can be found in online repositories. The names of the repository/repositories and accession number(s) can be found below: https://www.ncbi.nlm.nih.gov/nuccore/CP033505.1 and https://www.ncbi.nlm.nih.gov/nuccore/CP033506.1.

## Author Contributions

OR did the data processing and visualization, funding acquisition, manuscript writing and edition. IK did the sequencing, data processing and visualization, manuscript writing and edition. MJ did the data processing and visualization, manuscript writing and edition. SS did the sequencing, data processing and visualization. AJ did the microbiological procedures, data processing and visualization, manuscript writing and edition. NS did the microbiological procedures, data processing. AI did the project management and conceptualization, funding acquisition, manuscript writing and edition. All authors contributed to the article and approved the submitted version.

## Conflict of Interest

The authors declare that the research was conducted in the absence of any commercial or financial relationships that could be construed as a potential conflict of interest.

## Footnotes

1 https://rast.nmpdr.org/

2 https://card.mcmaster.ca/analyze

3 http://folk.uio.no/ohammer/past/

4 www.clinicaltrials.gov

## Supplementary Material

Supplementary Figure 1 Distribution of coverage values of m6A methylated sites identified on the chromosome of *S. aureus* BAA-39.

Supplementary Table 1 List of genes regulated at different conditions in the *S. aureus* ATCC BAA-39 NC and FS genomes.

Supplementary Table 2 List of BMF elements found in the *S. aureus* ATCC BAA-39 NC and FS genomes.

## Abbreviations

BM: base modification
BMF: bipartite methylated fragment
m4C: N^4^-methylcytosine
m6A: N^6^-methyladenine
MBC: minimal bactericidal concentration
modA: epigenetically modified adenine
modC: epigenetically modified cytosine
modG: epigenetically modified guanine
modT: epigenetically modified thymine
MRSA: methicillin-resistant *Staphylococcus aureus*
PacBio: Pacific Bioscience technology
SCC*mec*: staphylococcal methicillin-resistance chromosome cassette
SMRT: single molecule real-time sequencing.

## References

[B1] Adam M. Murali B. Glenn N. O. Potter S. S. (2008). Epigenetic inheritance based evolution of antibiotic resistance in bacteria. *BMC Evol. Biol.* 8:52. 10.1186/1471-2148-8-52 18282299 PMC2262874

[B2] Alcock B. P. Raphenya A. R. Lau T. T. Y. Tsang K. K. Bouchard M. Edalatmand A. (2020). CARD 2020: antibiotic resistome surveillance with the comprehensive antibiotic resistance database. *Nucleic Acids Res.* 48 D517–D525. 10.1093/nar/gkz935 31665441 PMC7145624

[B3] Almebairik N. Zamudio R. Ironside C. Joshi C. Ralph J. D. Roberts A. P. (2020). Genomic stability of composite SCCmec ACME and COMER-Like genetic elements in *Staphylococcus epidermidis* correlates with rate of excision. *Front. Microbiol.* 11:166. 10.3389/fmicb.2020.00166 32117176 PMC7029739

[B4] Andersson D. I. Hughes D. (2010). Antibiotic resistance and its cost: is it possible to reverse resistance? *Nat. Rev. Microbiol.* 8 260–271. 10.1038/nrmicro2319 20208551

[B5] Aziz R. K. Bartels D. Best A. A. DeJongh M. Disz T. Edwards R. A. (2008). The RAST Server: rapid annotations using subsystems technology. *BMC Genomics* 9:75. 10.1186/1471-2164-9-75 18261238 PMC2265698

[B6] Barbosa T. M. Levy S. B. (2000). The impact of antibiotic use on resistance development and persistence. *Drug Resist. Updat.* 3 303–311. 10.1054/drup.2000.0167 11498398

[B7] Baym M. Stone L. K. Kishony R. (2016). Multidrug evolutionary strategies to reverse antibiotic resistance. *Science* 351:aad3292. 10.1126/science.aad3292 26722002 PMC5496981

[B8] Bezuidt O. Lima-Mendez G. Reva O. N. (2009). SEQWord gene island sniffer: a program to study the lateral genetic exchange among bacteria. *World Acad. Sci. Eng. Technol.* 58 1169–1174.

[B9] Bierne H. Hamon M. Cossart P. (2012). Epigenetics and bacterial infections. *Cold Spring Harb. Perspect. Med.* 2:a010272. 10.1101/cshperspect.a010272 23209181 PMC3543073

[B10] Bigot S. Saleh O. A. Cornet F. Allemand J.-F. Barre F.-X. (2006). Oriented loading of FtsK on KOPS. *Nat. Struct. Mol. Biol.* 13 1026–1028. 10.1038/nsmb1159 17041597

[B11] Blackwell M. David C. Barker S. A. (2001). The presence of glycine betaine and the dextrinoid reaction in Basidiomata. *Harv. Pap. Bot.* 1 35–41.

[B12] Blow M. J. Clark T. A. Daum C. G. Deutschbauer A. M. Fomenkov A. Fries R. (2016). The epigenomic landscape of prokaryotes. *PLoS Genet.* 12:e1005854. 10.1371/journal.pgen.1005854 26870957 PMC4752239

[B13] Casadesús J. Low D. (2006). Epigenetic gene regulation in the bacterial world. *Microbiol. Mol. Biol. Rev.* 70 830–856. 10.1128/mmbr.00016-06 16959970 PMC1594586

[B14] Chaffin D. O. Taylor D. Skerrett S. J. Rubens C. E. (2012). Changes in the *Staphylococcus aureus* transcriptome during early adaptation to the lung. *PLoS One* 7:e41329. 10.1371/journal.pone.0041329 22876285 PMC3410880

[B15] Chaisson M. J. Tesler G. (2012). Mapping single molecule sequencing reads using basic local alignment with successive refinement (BLASR): application and theory. *BMC Bioinformatics* 13:238. 10.1186/1471-2105-13-238 22988817 PMC3572422

[B16] Chambers H. F. Deleo F. R. (2009). Waves of resistance: *Staphylococcus aureus* in the antibiotic era. *Nat. Rev. Microbiol.* 7 629–641. 10.1038/nrmicro2200 19680247 PMC2871281

[B17] Chin C. S. Alexander D. H. Marks P. Klammer A. A. Drake J. Heiner C. (2013). Nonhybrid, finished microbial genome assemblies from long-read SMRT sequencing data. *Nat. Methods* 10 563–569. 10.1038/nmeth.2474 23644548

[B18] Clatworthy A. E. Pierson E. Hung D. T. (2007). Targeting virulence: a new paradigm for antimicrobial therapy. *Nat. Chem. Biol.* 3 541–548. 10.1038/nchembio.2007.24 17710100

[B19] CLSI (2010). *Performance Standards for Antimicrobial Disk Susceptibility Test.* Available at: https://clsi.org/standards/products/microbiology/documents/m02/ (accessed January 30, 2010).

[B20] Contreras-Moreira B. Vinuesa P. (2013). GET_HOMOLOGUES, a versatile software package for scalable and robust microbial pangenome analysis. *Appl. Environ. Microbiol.* 79 7696–7701. 10.1128/aem.02411-13 24096415 PMC3837814

[B21] De Maeyer D. Renkens J. Cloots L. De Raedt L. Marchal K. (2013). PheNetic: network-based interpretation of unstructured gene lists in *E. coli*. *Mol. Biosyst.* 9 1594–1603. 10.1039/c3mb25551d 23591551

[B22] Dunai A. Spohn R. Farkas Z. Lázár V. Györkei Á. Apjok G. (2019). Rapid decline of bacterial drug-resistance in an antibiotic-free environment through phenotypic reversion. *eLife* 8:e47088. 10.7554/eLife.47088 31418687 PMC6707769

[B23] Dunner E. Brown W. B. Wallace J. (1949). The effect of streptomycin with para-amino salicylic acid on the emergence of resistant strains of tubercle bacilli. *Dis. Chest* 16 661–666. 10.1378/chest.16.6.661 15396513

[B24] Durão P. Balbontín R. Gordo I. (2018). Evolutionary mechanisms shaping the maintenance of antibiotic resistance. *Trends Microbiol.* 26 677–691. 10.1016/j.tim.2018.01.005 29439838

[B25] Ellis E. M. (2007). Reactive carbonyls and oxidative stress: potential for therapeutic intervention. *Pharmacol. Ther.* 115 13–24. 10.1016/j.pharmthera.2007.03.015 17570531

[B26] Epstein W. (1986). Osmoregulation by potassium transport in *Escherichia coli*. *FEMS Microbiol. Rev.* 39 73–78. 10.1111/j.1574-6968.1986.tb01845.x

[B27] Ezraty B. Gennaris A. Barras F. Collet J. F. (2017). Oxidative stress, protein damage and repair in bacteria. *Nat. Rev. Microbiol.* 15 385–396. 10.1038/nrmicro.2017.26 28420885

[B28] Falkenberg P. Strøm A. R. (1990). Purification and characterization of osmoregulatory betaine aldehyde dehydrogenase of *Escherichia coli*. *Biochim. Biophys. Acta* 1034 253–259. 10.1016/0304-4165(90)90046-y 2194570

[B29] Fang C. T. Yi W. C. Shun C. T. Tsai S. F. (2017). DNA adenine methylation modulates pathogenicity of *Klebsiella pneumoniae* genotype K1. *J. Microbiol. Immunol. Infect.* 50 471–477. 10.1016/j.jmii.2015.08.022 26427879

[B30] Foti J. J. Devadoss B. Winkler J. A. Collins J. J. Walker G. C. (2012). Oxidation of the guanine nucleotide pool underlies cell death by bactericidal antibiotics. *Science* 336 315–319. 10.1126/science.1219192 22517853 PMC3357493

[B31] Freeman J. M. Plasterer T. N. Smith T. F. Mohr S. C. (1998). Patterns of genome organization in bacteria. *Science* 279:1827 10.1126/science.279.5358.1827a

[B32] Fuchs S. Pané-Farré J. Kohler C. Hecker M. Engelmann S. (2007). Anaerobic gene expression in *Staphylococcus aureus*. *J. Bacteriol.* 189 4275–4289. 10.1128/jb.00081-07 17384184 PMC1913399

[B33] Golosova O. Henderson R. Vaskin Y. Gabrielian A. Grekhov G. Nagarajan V. (2014). Unipro UGENE NGS pipelines and components for variant calling, RNA-seq and ChIP-seq data analyses. *PeerJ* 2:e644. 10.7717/peerj.644 25392756 PMC4226638

[B34] Gründling A. (2013). Potassium uptake systems in *Staphylococcus aureus*: new stories about ancient systems. *mBio* 4:e00784-13. 10.1128/mBio.00784-13 PMC3791899 24105767

[B35] Gu S. Xiong J. Shi Y. You J. Zou Z. Liu X. (2017). Error-prone bypass of O6-methylguanine by DNA polymerase of *Pseudomonas aeruginosa* phage PaP1. *DNA Repair* 57 35–44. 10.1016/j.dnarep.2017.06.021 28651167

[B36] Haagmans W. van der Woude M. (2000). Phase variation of Ag43 in *Escherichia coli*: dam-dependent methylation abrogates OxyR binding and OxyR-mediated repression of transcription. *Mol. Microbiol.* 35 877–887. 10.1046/j.1365-2958.2000.01762.x 10692164

[B37] Hammer Ø. Harper D. A. Ryan P. D. (2001). PAST: paleontological statistics software package for education and data analysis. *Palaeontol. Electron.* 4:9.

[B38] Harhay G. P. Harhay D. M. Bono J. L. Capik S. F. DeDonder K. D. Apley M. D. (2019). A computational method to quantify the effects of slipped strand mispairing on bacterial tetranucleotide repeats. *Sci. Rep.* 9:18087. 10.1038/s41598-019-53866-z 31792233 PMC6889271

[B39] Henderson I. R. Owen P. Nataro J. P. (1999). Molecular switches – the ON and OFF of bacterial phase variation. *Mol. Microbiol.* 33 919–932. 10.1046/j.1365-2958.1999.01555.x 10476027

[B40] Hendrickson H. L. Barbeau D. Ceschin R. Lawrence J. G. (2018). Chromosome architecture constrains horizontal gene transfer in bacteria. *PLoS Genet.* 14:e1007421. 10.1371/journal.pgen.1007421 29813058 PMC5993296

[B41] Hernday A. Krabbe M. Braaten B. Low D. (2002). Self-perpetuating epigenetic pili switches in bacteria. *Proc. Natl. Acad. Sci. U.S.A.* 99(Suppl. 4) 16470–16476. 10.1073/pnas.182427199 12202745 PMC139910

[B42] Hershberg R. (2015). Mutation - the engine of evolution: studying mutation and its role in the evolution of bacteria. *Cold Spring Harb. Perspect. Biol.* 7:a018077. 10.1101/cshperspect.a018077 26330518 PMC4563715

[B43] Holden M. T. Feil E. J. Lindsay J. A. Peacock S. J. Day N. P. Enright M. C. (2004). Complete genomes of two clinical *Staphylococcus aureus* strains: evidence for the rapid evolution of virulence and drug resistance. *Proc. Natl. Acad. Sci. U.S.A.* 101 9786–9791. 10.1073/pnas.0402521101 15213324 PMC470752

[B44] Hollinshead W. D. Rodriguez S. Martin H. G. Wang G. Baidoo E. E. Sale K. L. (2016). Examining *Escherichia coli* glycolytic pathways, catabolite repression, and metabolite channeling using Δ*pfk* mutants. *Biotechnol. Biofuels* 9:212. 10.1186/s13068-016-0630-y 27766116 PMC5057261

[B45] Ilin A. I. Kulmanov M. E. (2014). Antibacterial agent for treating infectious diseases of bacterial origin. U.S. Patent No 20,140,010,782. Washington, DC: U.S. Patent and Trademark Office.

[B46] Ilin A. I. Kulmanov M. E. Korotetskiy I. S. Akhmetova G. K. Lankina M. V. Shvidko S. V. (2015). Complete genome sequence of multidrug-resistant clinical isolate *Mycobacterium tuberculosis* 187.0, used to study the effect of drug susceptibility reversion by the new medicinal drug FS-1. *Genome Announc*. 3:e01272-15. 10.1128/genomeA.01272-15 PMC4645197 26543112

[B47] Ilin A. I. Kulmanov M. E. Korotetskiy I. S. Islamov R. A. Akhmetova G. K. Lankina M. V. (2017). Genomic insight into mechanisms of reversion of antibiotic resistance in multidrug resistant *Mycobacterium tuberculosis* induced by a nanomolecular iodine-containing complex FS-1. *Front. Cell. Infect. Microbiol.* 7:151. 10.3389/fcimb.2017.00151 28534009 PMC5420568

[B48] Ilin A. I. Parsadanyan G. G. Nersesyan A. K. (2013). Interaction of halogens with nucleic acids and its consequence. *New Armen. Med. J.* 3 33–43.

[B49] Islamov R. Kerimzhanova B. Ilin A. (2018). “New antituberculosis drug FS-1,” in *Medicinal Chemistry*, eds Vašková J. Vaško L. (London: IntechOpen), 103–116.

[B50] Jani M. Sengupta S. Hu K. Azad R. K. (2017). Deciphering pathogenicity and antibiotic resistance islands in methicillin-resistant *Staphylococcus aureus* genomes. *Open Biol.* 7:170094. 10.1098/rsob.170094 29263245 PMC5746543

[B51] Joubert M. Reva O. N. Korotetskiy I. S. Shvidko S. V. Shilov S. V. Jumagaziyeva A. B. (2019). Assembly of complete genome sequences of negative-control and experimental strain variants of *Staphylococcus aureus* ATCC BAA-39 selected under the effect of the drug FS-1, which induces antibiotic resistance reversion. *Microbiol. Resour. Announc.* 8:e00579-19. 10.1128/MRA.00579-19 PMC6658690 31346020

[B52] Kohler C. von Eiff C. Liebeke M. McNamara P. J. Lalk M. Proctor R. A. (2008). A defect in menadione biosynthesis induces global changes in gene expression in *Staphylococcus aureus*. *J. Bacteriol.* 190 6351–6364. 10.1128/jb.00505-08 18676673 PMC2566006

[B53] Korotetskiy I. S. Joubert M. Magabotha S. Jumagaziyeva A. B. Shilov S. V. Suldina N. A. (2020a). Complete genome sequence of a collection strain *Acinetobacter baumannii* ATCC BAA-1790 used as a model to study the antibiotic resistance reversion induced by the iodine-containing complexes. *Microbiol. Resour. Announc.* 9:e01467-19. 10.1128/MRA.01467-19 PMC6965589 31948971

[B54] Korotetskiy I. S. Joubert M. Taukobong S. Jumagaziyeva A. B. Shilov S. V. Shvidko S. V. (2019a). Complete genome sequence of a multidrug-resistant strain, *Escherichia coli* ATCC BAA-196, as a model for studying induced antibiotic resistance reversion. *Microbiol. Resour. Announc.* 8:e01118-19. 10.1128/MRA.01118-19 PMC6908795 31831610

[B55] Korotetskiy I. S. Jumagaziyeva A. B. Reva O. N. Kuznetsova T. V. Shvidko S. V. Iskakbayeva Z. A. (2019b). Isolation and characterization isolates of nosocomial infections. *Bull. Nat. Acad. Sci. Rep. Kazakhstan* 5 199–209. 10.32014/2019.2518-1467.140

[B56] Korotetskiy I. S. Jumagaziyeva A. B. Shilov S. V. Kuznetsova T. V. Suldina N. A. Kenesheva S. T. (2020b). Differential gene expression and alternation of patterns of DNA methylation in the multidrug resistant strain *Escherichia coli* ATCC BAA-196 caused by iodine-containing nano-micelle drug FS-1 that induces antibiotic resistance reversion. *bioRxiv [Preprint]* 10.1101/2020.05.15.097816

[B57] Korotetskiy I. S. Shilov S. V. Shvidko S. V. Jumagaziyeva A. B. Suldina N. A. Korotetskaya N. V. (2017). Transcriptional response of the multidrug resistant *Staphylococcus aureus* following FS-1 exposure. *Eurasian J. Appl. Biotechnol.* 2017 43–48. 10.11134/btp.3.2017.6

[B58] Kosmachevskaya O. V. Shumaev K. B. Topunov A. F. (2015). Carbonyl stress in bacteria: causes and consequences. *Biochemistry* 80 1655–1671. 10.1134/S0006297915130039 26878572

[B59] Kumar O. R. Singh B. R. Sinha K. Dubal Z. B. Pruthvishree B. S. Rupner R. N. (2019). Tackling antimicrobial resistance: current approaches. *J. Immun. Immunopathol.* 21 1–9. 10.5958/0973-9149.2019.00001.7

[B60] Lin W. Zeng J. Wan K. Lv L. Guo L. Li X. (2018). Reduction of the fitness cost of antibiotic resistance caused by chromosomal mutations under poor nutrient conditions. *Environ. Int.* 120 63–71. 10.1016/j.envint.2018.07.035 30064056

[B61] Liu L. Zhang Y. Jiang D. Du S. Deng Z. Wang L. (2019). Recent advances in the genomic profiling of bacterial epigenetic modifications. *Biotechnol. J.* 14:e1800001. 10.1002/biot.201800001 29878585

[B62] Liu X.-Y. Zhu M.-X. Xie J.-P. (2010). Mutagenicity of acrolein and acrolein-induced DNA adducts. *Toxicol. Mech. Meth.* 20 36–44. 10.3109/15376510903530845 20158384

[B63] Love M. I. Huber W. Anders S. (2014). Moderated estimation of fold change and dispersion for RNA-seq data with DESeq2. *Genome Biol.* 15:550. 10.1186/s13059-014-0550-8 PMC4302049 25516281

[B64] Low D. A. Casadesús J. (2008). Clocks and switches: bacterial gene regulation by DNA adenine methylation. *Curr. Opin. Microbiol.* 11 106–112. 10.1016/j.mib.2008.02.012 18396448

[B65] Marczynski G. T. Shapiro L. (1993). Bacterial chromosome origins of replication. *Curr. Opin. Genet. Dev.* 3 775–782. 10.1016/s0959-437x(05)80098-x 8274862

[B66] Marinus M. G. Løbner-Olesen A. (2014). DNA methylation. *EcoSal Plus* 6 607–617. 10.1128/ecosalplus.ESP-0003-2013 PMC4231299 26442938

[B67] Neeley W. L. Essigmann J. M. (2006). Mechanisms of formation, genotoxicity, and mutation of guanine oxidation products. *Chem. Res. Toxicol.* 19 491–505. 10.1021/tx0600043 16608160

[B68] Nigam A. Gupta D. Sharma A. (2014). Treatment of infectious disease: beyond antibiotics. *Microbiol. Res.* 169 643–651. 10.1016/j.micres.2014.02.009 24661689

[B69] Nyce J. Leonard S. Canupp D. Schulz S. Wong S. (1993). Epigenetic mechanisms of drug resistance: drug-induced DNA hypermethylation and drug resistance. *Proc. Natl. Acad. Sci. U.S.A.* 90 2960–2964. 10.1073/pnas.90.7.2960 8464912 PMC46216

[B70] O’Dell K. B. Hatmaker E. A. Guss A. M. Mormile M. R. (2018). Complete genome sequence of *Salinisphaera* sp. strain LB1, a moderately halo-acidophilic bacterium isolated from Lake Brown, Western Australia. *Microbiol. Resour. Announc*. 7:e01047-18. 10.1128/MRA.01047-18 PMC6256560 30533691

[B71] Phillips Z. N. Husna A. U. Jennings M. P. Seib K. L. Atack J. M. (2019a). Phasevarions of bacterial pathogens–phase-variable epigenetic regulators evolving from restriction–modification systems. *Microbiology* 165 917–928. 10.1099/mic.0.000805 30994440

[B72] Phillips Z. N. Tram G. Seib K. L. Atack J. M. (2019b). Phase-variable bacterial loci: how bacteria gamble to maximise fitness in changing environments. *Biochem. Soc. Trans.* 47 1131–1141. 10.1042/BST20180633 31341035

[B73] Poulikakos P. Tansarli G. S. Falagas M. E. (2014). Combination antibiotic treatment versus monotherapy for multidrug-resistant, extensively drug-resistant, and pandrug-resistant *Acinetobacter* infections: a systematic review. *Eur. J. Clin. Microbiol. Infect. Dis.* 33 1675–1685. 10.1007/s10096-014-2124-9 24832022

[B74] Richarme G. Liu C. Mihoub M. Abdallah J. Leger T. Joly N. (2017). Guanine glycation repair by DJ-1/Park7 and its bacterial homologs. *Science* 357 208–211. 10.1126/science.aag1095 28596309

[B75] Roberts R. J. Carneiro M. O. Schatz M. C. (2013). The advantages of SMRT sequencing. *Genome Biol.* 14:405. 10.1186/gb-2013-14-7-405 PMC3953343 23822731

[B76] Roberts R. J. Vincze T. Posfai J. Macelis D. (2015). REBASE – a database for DNA restriction and modification: enzymes, genes and genomes. *Nucleic Acids Res.* 43 D298–D299. 10.1093/nar/gku1046 25378308 PMC4383893

[B77] Rocha-Granados M. C. Zenick B. Englander H. E. Mok W. W. K. (2020). The social network: impact of host and microbial interactions on bacterial antibiotic tolerance and persistence. *Cell. Signal.* 75:109750. 10.1016/j.cellsig.2020.109750 32846197

[B78] Roux D. Danilchanka O. Guillard T. Cattoir V. Aschard H. Fu Y. (2015). Fitness cost of antibiotic susceptibility during bacterial infection. *Sci. Transl. Med.* 7:297ra114. 10.1126/scitranslmed.aab1621 26203082

[B79] Sánchez-Romero M. A. Casadesús J. (2020). The bacterial epigenome. *Nat. Rev. Microbiol.* 18 7–20. 10.1038/s41579-019-0286-2 31728064

[B80] Sánchez-Romero M. A. Cota I. Casadesús J. (2015). DNA methylation in bacteria: from the methyl group to the methylome. *Curr. Opin. Microbiol.* 25 9–16. 10.1016/j.mib.2015.03.004 25818841

[B81] Sarker M. R. Islam K. N. Huri H. Z. Imam H. Hosen B. Rahman M. (2014). Studies of the impact of occupational exposure of pharmaceutical workers on the development of antimicrobial drug resistance. *J. Occup. Health* 56 260–270. 10.1539/joh.14-0012-OA 24953094

[B82] Semchyshyn H. M. Lushchak V. I. (2012). “Interplay between oxidative and carbonyl stresses: molecular mechanisms, biological effects and therapeutic strategies of protection,” in *Oxidative Stress - Molecular Mechanisms and Biological Effects*, ed. Lushchak V. I. (London: IntechOpen), 15–46.

[B83] Simão F. A. Waterhouse R. M. Ioannidis P. Kriventseva E. V. Zdobnov E. M. (2015). BUSCO: assessing genome assembly and annotation completeness with single-copy orthologs. *Bioinformatics* 31 3210–3212. 10.1093/bioinformatics/btv351 26059717

[B84] Sitthisak S. Knutsson L. Webb J. W. Jayaswal R. K. (2007). Molecular characterization of the copper transport system in *Staphylococcus aureus*. *Microbiology* 153 4274–4283. 10.1099/mic.0.2007/009860-0 18048940

[B85] Sjolund M. Tano E. Blaser M. J. Andersson D. I. Engstrand L. (2005). Persistence of resistant *Staphylococcus epidermidis* after single course of clarithromycin. *Emerg. Infect. Dis.* 11 1389–1393. 10.3201/eid1109.050124 16229767 PMC3310621

[B86] Sjolund M. Wreiber K. Andersson D. I. Blaser M. J. Engstrand L. (2003). Long-term persistence of resistant *Enterococcus* species after antibiotics to eradicate *Helicobacter pylori*. *Ann. Intern. Med.* 139 483–487. 10.7326/0003-4819-139-6-200309160-00011 13679325

[B87] Spellberg B. Powers J. H. Brass E. P. Miller L. G. Edwards J. E. Jr. (2004). Trends in antimicrobial drug development: implications for the future. *Clin. Infect. Dis.* 38 1279–1286. 10.1086/420937 15127341

[B88] Stojanov M. Moreillon P. Sakwinska O. (2015). Excision of staphylococcal cassette chromosome *mec* in methicillin-resistant *Staphylococcus aureus* assessed by quantitative PCR. *BMC Res. Notes* 8:828. 10.1186/s13104-015-1815-3 26715147 PMC4693430

[B89] Sullivan M. J. Altman D. R. Chacko K. I. Ciferri B. Webster E. Pak T. R. (2019). A complete genome screening program of clinical methicillin-resistant *Staphylococcus aureus* isolates identifies the origin and progression of a neonatal intensive care unit outbreak. *J. Clin. Microbiol.* 57:e01261-19. 10.1128/JCM.01261-19 PMC6879278 31578260

[B90] Tenover F. C. (2001). Development and spread of bacterial resistance to antimicrobial agents: an overview. *Clin. Infect. Dis.* 33(Suppl. 3) S108–S115. 10.1086/321834 11524705

[B91] van der Woude M. W. Bäumler A. J. (2004). Phase and antigenic variation in bacteria. *Clin. Microbiol. Rev.* 17 581–611. 10.1128/CMR.17.3.581-611.2004 15258095 PMC452554

[B92] van Niekerk K. Pierneef R. Reva O. N. Korostetskiy I. S. Ilin A. I. Akhmetova G. K. (2018). “Clade-specific distribution of antibiotic resistance mutations in the population of *Mycobacterium tuberculosis* – prospects of drug resistance reversion,” in *Basic Biology and Applications of Actinobactera*, ed. Enany S. (London: IntechOpen), 79–98. 10.5772/intechopen.75181

[B93] Ventola C. L. (2015). The antibiotic resistance crisis: part 1: causes and threats. *P T* 40 277–283. 25859123 PMC4378521

[B94] Vitko N. P. Richardson A. R. (2013). Laboratory maintenance of methicillin-resistant *Staphylococcus aureus* (MRSA). *Curr. Protoc. Microbiol.* 28 9C.2.1–9C.2.14. 10.1002/9780471729259.mc09c02s28 23408135 PMC4070006

[B95] Wall S. (2019). Prevention of antibiotic resistance - an epidemiological scoping review to identify research categories and knowledge gaps. *Glob. Health Action* 12:1756191. 10.1080/16549716.2020.1756191 32475304 PMC7782542

[B96] Wang J. Foxman B. Mody L. Snitkin E. S. (2017). Network of microbial and antibiotic interactions drive colonization and infection with multidrug-resistant organisms. *Proc. Natl. Acad. Sci. U.S.A.* 114 10467–10472. 10.1073/pnas.1710235114 28900004 PMC5625923

[B97] Zhang X. Xu X. Yuan W. Hu Q. Shang W. Hu X. (2014). Complete genome sequence of *Staphylococcus aureus* XN108, an ST239-MRSA-SCC*mec* III strain with intermediate vancomycin resistance isolated in mainland China. *Genome Announc.* 2:e00449-14. 10.1128/genomeA.00449-14 PMC4110214 25059856

[B98] Zhu X. Radovic-Moreno A. F. Wu J. Langer R. Shi J. (2014). Nanomedicine in the management of microbial infection - overview and perspectives. *Nano Today* 9 478–498. 10.1016/j.nantod.2014.06.003 25267927 PMC4175422

