# Supplementary figures and images for: The Effect of Iodine-Containing Nano-Micelles, FS-1, on Antibiotic Resistance, Gene Expression and Epigenetic Modifications in the Genome of Multidrug Resistant MRSA Strain Staphylococcus aureus ATCC BAA-39
Source: Front Microbiol. 2020 Oct 22;11:581660. doi: 10.3389/fmicb.2020.581660 (PMC7642360; doi:10.3389/fmicb.2020.581660)

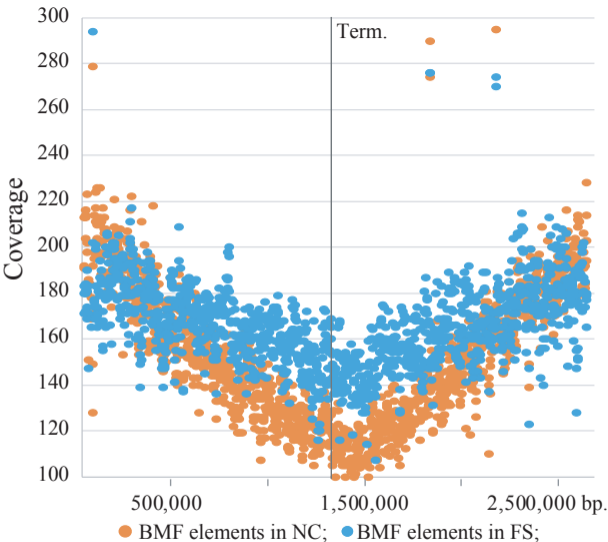

**Figure S1** | Distribution of coverage values in BMF elements.

Supplement: Supplementary Figure 1 — Distribution of coverage values of m6A methylated sites identified on the chromosome of S. aureus BAA-39. [file Data_Sheet_1.PDF]
